# Nosocomial dissemination of hypervirulent *Klebsiella pneumoniae* with high-risk clones among children in Shanghai

**DOI:** 10.3389/fcimb.2022.984180

**Published:** 2022-08-29

**Authors:** Qingqing Du, Fen Pan, Chun Wang, Fangyuan Yu, Yingying Shi, Wenxin Liu, Zhi Li, Ping He, Dingding Han, Hong Zhang

**Affiliations:** ^1^ Department of Clinical Laboratory, Shanghai Children’s Hospital, School of Medicine, Shanghai Jiao Tong University, Shanghai, China; ^2^ Department of Pathology, Shanghai Children’s Hospital, School of Medicine, Shanghai Jiao Tong University, Shanghai, China; ^3^ Department of Medical Microbiology and Immunology, School of Medicine, Shanghai Jiao Tong University, Shanghai, China

**Keywords:** *Klebsiella pneumoniae*, hypervirulence, carbapenem-resistant, child, infection, surveillance

## Abstract

**Objectives:**

Although hypervirulent *Klebsiella pneumoniae* (hvKp) is an increasing public health problem, there remains limited epidemiological information regarding hvKp infections in children. Here, we conducted a clinical, molecular and phenotypic surveillance of hvKp strains in a pediatric population.

**Methods:**

Non-repetitive *K. pneumoniae* (Kp) strains consecutively collected during 2019-2020 were screened for hypervirulence genes (*prmpA*, *prmpA2*, *iucA*, *iroB*, and *peg344*) using PCR. Positive strains were further characterized by four phenotypic assays (string test, serum killing assay, siderophore production, *Galleria mellonella* lethality assay), followed by murine sepsis model to determine virulence *in vitro* and *in vivo*. Also, capsular types, sequence types, plasmid replicon types, antimicrobial resistance determinants and susceptibility were analyzed.

**Results:**

A total of 352 isolates were collected, wherein 83 (23.6%) were hypervirulence genes-positive Kp (hgKp). A significant increase in KPC-2-producing KL47-ST11 among hgKp strains was observed, from 5.3% (1/19) in 2019 to 67.6% (25/37) in 2020 (*P*<.0001), suggesting the potential dissemination of the hybrid virulence and carbapenem-resistance encoding plasmid among children. Further, hgKp isolates were classified into hvKp (n = 27) and hgKp-low virulence (hgKp-Lv) (n = 56) based on virulence phenotypic assays. In hvKp, diverse genetic clones were observed and K1-ST23 or K2-ST25 strains with sensitivity to multiple antibiotics were prevalent (25.9%, 7/27). Compared with hgKp-Lv, hvKp infection had a higher propensity to involve severe pneumonia (22.2% vs. 12.5%) in elder children and significant higher mortality in mice (*P* = 0.0086). Additionally, either hvKp or hgKp-Lv infections were mostly healthcare-associated and hospital-acquired (74.1% vs. 91.9%).

**Conclusions:**

These data suggest that K1-ST23 and K2-ST25 are high-risk clones of hvKp, and the genetic convergence of virulence and carbapenem-resistance is increasing among children. Control measures are needed to prevent the dissemination in clinical settings.

## Introduction

*Klebsiella pneumoniae* is a common opportunistic pathogen encountered in clinical practice. Hypervirulent *K. pneumoniae* (hvKp), a virulent variant of ‘classical’ *K. pneumoniae* (cKp), has become a global public health problem (Turton et al., 2007; Siu et al., 2011). Although cKp is a leading cause of nosocomial infections worldwide, the more destructive hvKp variant can cause community-acquired infections in healthy individuals; these infections eventually affect distant sites (e.g., eyes, lungs, and central nervous system), resulting in high morbidity and mortality (Wang et al., 2005; Prokesch et al., 2016). Importantly, the convergence of hypervirulence in hvKp and carbapenem resistance in carbapenem-resistant Kp (CRKp) has led to a fatal outbreak in China (Gu et al., 2018). Recently, carbapenem-resistant hvKp (CR-hvKp) has emerged as a global pathogen (Karlsson et al., 2019; Wozniak et al., 2019).

The hypervirulence of hvKp is attributed to characteristics such as its capsule, lipopolysaccharide, types 1 and 3 fimbriae, siderophores, and allantoin metabolism (Paczosa and Mecsas, 2016); these characteristics are controlled by large virulence plasmids and integrated chromosomal elements. Thus, hvKp strains can be identified using genetic biomarkers such as *prmpA/A2* (capsule production regulator), *iuc* (aerobactin synthesis), *iro* (salmochelin biosynthesis), and *peg344* (a metabolic transporter of unknown function) (Lam et al., 2018; Russo et al., 2018). Hypermucoviscosity is also a characteristic phenotype of many hvKp isolates. However, the presence of virulence genes or the hypermucoviscous phenotype alone is not a consistent indicator of hypervirulence *in vivo* (Russo et al., 2018). Therefore, previous reports of hvKp based on these screening modalities may not accurately represent the epidemiological trends of such strains. A combination of hypervirulence gene screening and virulence phenotype identification is needed to distinguish between strains of hvKp and strains of cKp.

Thus far, studies of hvKp have mainly focused on adults except the most recent surveillance performed between 2017 and 2019, which indicates that there is an increasing risk of hvKp infections in children (Li et al., 2021). To gain a comprehensive understanding of current hvKp epidemiology in children, we evaluated the clinical, molecular, and phenotypic characteristics of hvKp isolates from infected children in Shanghai, China.

## Materials and methods

### Patients and specimens

In total, 352 consecutive non-replicate clinical *K. pneumoniae* (Kp) isolates were recovered from Kp culture-positive patients at Shanghai Children’s Hospital from January 2019 to December 2020, including 168 sputum samples, 62 urine samples, 58 catheter samples, 15 blood samples, 15 sterile body fluid samples, 15 pus samples and 19 other samples. Collection of blood specimens was performed according to the Clinical and Laboratory Standard Institute (CLSI) (CLSI, 2007). Shortly, every two bottles, containing 2-6 ml the left and the right side of venous blood from every patient, respectively, were incubated in a BACTEC FX200-automated blood culture system (BD Biosciences, America) and were monitored for up to 7 days.

Clinical information was systematically extracted from the patients’ medical records. In this study, nosocomial infections were defined as infections that developed more than 48 hours after hospital admission. Healthcare-associated infections were defined as infections in patients who had interacted with the healthcare system prior to the onset of infection (e.g., receipt of intravenous chemotherapy within 30 days, hospitalization in an acute care hospital within 90 days, or residence in a nursing home or long-term-care facility). Community-acquired infections were defined as infections that developed within 48 h of admission in patients who did not fulfill the criteria for a healthcare-associated infection (Friedman et al., 2002).

### Bacterial identification and antimicrobial susceptibility testing

All strains were identified by MALDI-TOF mass spectrometry (Bruker Diatonic GmbH, Germany). Antimicrobial susceptibility was confirmed by Kirby-Bauer disk and VITEK 2 system tests. Susceptibility tests were performed, and results were interpreted, in accordance with guidelines from the CLSI (CLSI, 2021). The *in vitro* activity of tigecycline was determined using standards from the Food and Drug Administration (CDER, 2005), while the *in vitro* activity of polymyxin B was determined using thresholds from the European Committee on Antimicrobial Susceptibility Testing (Tsuji et al., 2019). The quality control strain was *Escherichia coli* ATCC 25922.

Multi-drug resistance was defined as acquired non-susceptibility to at least one agent in three or more antimicrobial categories according to Magiorakos et al. (Magiorakos et al., 2012); CRKp was regarded as strains with resistance to ertapenem, imipenem, or meropenem.

### Molecular methods

All isolates were screened by PCR assays that targeted *prmpA, prmpA2, iucA, iroB*, or *peg344* hypervirulence genes. Initially, we used a previously described multiplex-PCR method (Yu et al., 2018), then confirmed positive strains by separate singleplex-PCR assays for each gene (Russo et al., 2018). Additionally, we used ‘*prmpA*’ and ‘*aerobactin*’ primers to review the genotypes of plasmid-derived *prmpA*/*A2* and ‘*iucA*’ genes, respectively (**Table S1**). The hypervirulent strain NTUH-K2044 was used as a positive control. Each gene detection assay was performed in duplicate and repeated three times independently.

For all hypervirulence genes-positive *K. pneumoniae* (hgKp) isolates, antimicrobial resistance genes were amplified by PCR; the PCR amplicons of carbapenemase genes were sequenced. Plasmid replicons were typed using a PCR-based protocol, as previously described (Carattoli et al., 2005). Common hypervirulent capsular serotypes K1, K2, K5, K20, K54, K57 and common carbapenem-resistant capsular serotypes KL47, KL64 were identified by PCR and sequencing, and other capsular serotypes were determined by *wzi* gene amplification and sequencing. Multilocus sequences were determined by sequencing seven housekeeping genes; subsequent homology analysis was performed on the basis of fingerprinting profiles from Enterobacterial Repetitive Intergenic Consensus-Polymerase Chain Reaction (ERIC-PCR) (**Table S1**).

### Phenotypic assays

The hypermucoviscous phenotype was defined by a positive string test result (a viscous string > 5 mm in length) (Fang et al., 2004).

Serum resistance was determined using a previously described method (Podschun et al., 1991). Briefly, serum was obtained from healthy individuals and stored at 280°C. Exponential-phase phase bacterial at 1 × 10^6^ CFU/ml were mixed at a 1:3 ratios with healthy human serum, and the mixtures were incubated at 37°C with shaking. Viable counts were obtained at 0, 1, 2, and 3 h, according to bacterial counts at these time points, the response of strains to serum killing was scored six grades, namely serum-sensitive (grade 1 or 2), intermediately sensitive (grade 3 or 4), or serum-resistant (grade 5 or 6). For grade 1, viable counts were <10% of the inoculum at 1, 2 h and <0.1% at 3 h. For grade 2, viable counts were 10-100% of the inoculum at 1 h and <10% at 3 h. For grade 3, viable counts were >100% of inoculum at 1 h and <100% at 2, 3 h. For grade 4, viable counts were >100% of inoculum at 1, 2 h and <100% at 3 h. For grade 5, viable counts were >100% of the inoculum at 1, 2, and 3 h, but decreased during the 3-hour period. For grade 6, viable counts >100% of the inoculum at 1, 2, and 3 h, and increased throughout the time period. Each experiment was performed in triplicate.

Relative quantitative siderophore production was measured using the method of Russo et al. and Tian et al. (Russo et al., 2018; Tian et al., 2021): a colony of strains was grown overnight in iron-chelated M9 minimal medium containing Casamino Acids (c-M9-CA) at 37°C, 180rpm. 100ul of supernatant of each bacterial suspension which was diluted 5-fold in c-M9-CA and 100 ul of CAS solution were added to a flat-bottom 96-well plate, after incubation in the dark for 30 min, and were measured at A630 nm. The c-M9-CA was plus CAS solution was used as a reference. Siderophore units (Su) were defined by the ratio of samples absorbance (As) to reference absorbance (Ar), namely [(Ar – As)/Ar] × 100 = X%. The hypervirulent strain NTUH-K2044 and the cKp (randomly selected one strain from CRKp subset without hypervirulence gene) were used as hypervirulence and low-virulence controls, respectively. Each assay was performed in duplicate and repeated in triplicate independently.

A *Galleria mellonella* larvae infection model was established as previously described (Zhang et al., 2020): *G. mellonella* larvae (pathogen-free, 250-350mg) were obtained from Huiyude Biotech Company (Tianjin, China), ten per group, were inoculated with 10 μL of exponential-phase bacterial suspension (at a concentration of 1 × 10^7^ CFU/mL) into the left proleg using a micro-sample syringe. Larvae in petri dishes were incubated at 37C in the dark for 72 h to observe mortality rates. Strains NTUH-K2044 and cKp were used as the respective hypervirulence and low-virulence controls. Each experiment was performed in triplicate.

Mouse lethality assays were performed using 5-week-old male BALB/c mice (mean weight, approximately 16 g) from Shanghai JieSiJie Laboratory Animal Co., Ltd. (Shanghai, China). For each isolate, five mice per group were intraperitoneally infected with bacteria of 1×10^6^ CFU. The sample size was estimated using 90% power at an alpha level of 0.5 (https://www.bu.edu/researchsupport/compliance/animal-care/working-with-animals/research/sample-size-calculations-iacuc/). All mice were subjected to mortality monitoring for 7 days. Within 12 hours of a mouse’s death, its organs (heart, liver, lungs, and kidneys) were aseptically harvested and homogenized to plate on LB agar with serial dilutions for CFU enumeration. Organ sections were fixed in paraformaldehyde and embedded in paraffin, then subjected to H&E and Gram staining to assess areas of bleeding. For each organ sample from septic mice, two sections were randomly chosen. Areas of bleeding were evaluated in four different views of each section, using ImageJ software (National Institutes of Health, USA). Strains NTUH-K2044 and cKp were used as the respective hypervirulence and low-virulence controls.

### Determination of hgKp, hvKp and hgKp-Lv

The hypervirulence genes *prmpA*, *prmpA2*, *iucA*, *iroB* and *peg344* are biomarkers with high accuracy for identifying hvKp strains (Russo et al., 2018). In this study, *K. pneumoniae* carrying these five genes was termed as hgKp, which could be classified into hvKp and hgKp-low virulence (hgKp-Lv). HvKp was defined by the presence of at least one hypervirulence gene, as well as the high lethality in *G. mellonella* and at least one other hypervirulence phenotype in the string test, serum killing assay, and/or relative quantitative siderophore production assay. Non-hvKp with hypervirulence gene(s) was termed as hgKp-Lv.

### Statistical analysis

All statistical analyses were performed using SAS version 8.0. Categorical variables are shown as frequencies with percentages; they were analyzed using the chi-squared test or Fisher’s exact test. Continuous data are shown as means ± standard deviations or medians (interquartile ranges); they were analyzed using independent-samples t-tests or the Mann–Whitney U test. *P*-values < 0.05 were considered statistically significant.

## Results

### Identification of hypervirulent *K. pneumoniae*

**Figure 1:**
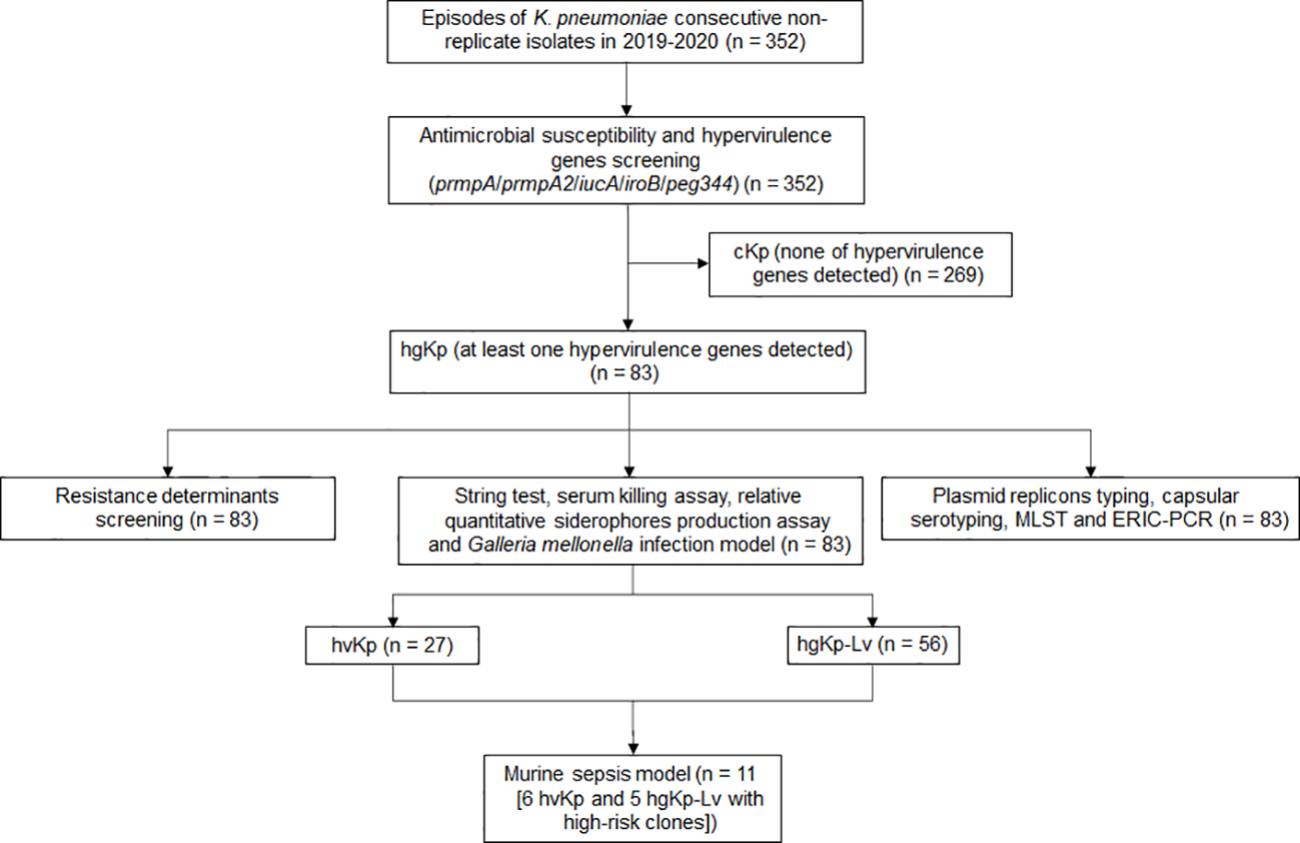
Flowchart for screening hypervirulent *K. pneumoniae* (hvKp) in this study. HgKp, hypervirulence genes-positive *K. pneumoniae*; hgKp-Lv, hgKp-low virulence.

**Figure 2:**
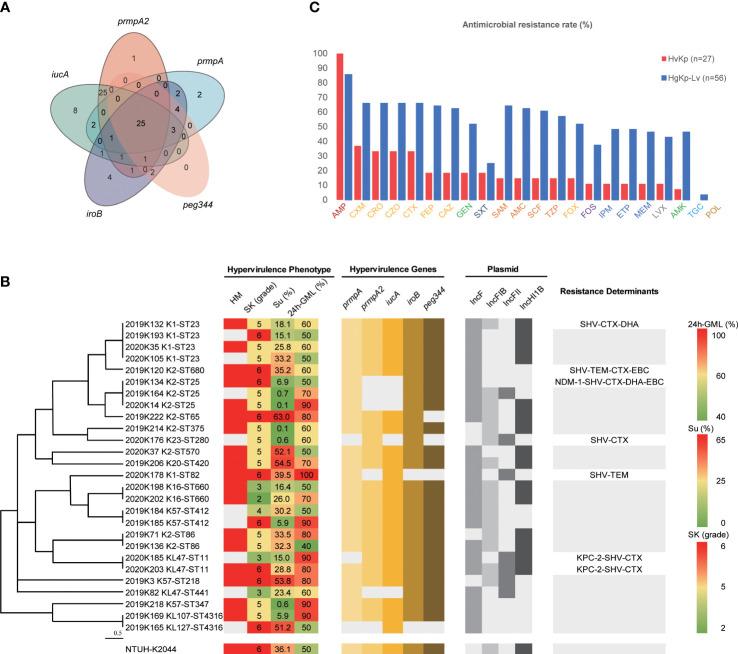
Features of hypervirulent *K pneumoniae* strains. **(A)** Distribution of hypervirulence genes among hgKp isolates (n = 83). **(B)** Hypervirulence genes, hypervirulence phenotypes, plasmid types, and resistance genes of hvKp isolates (n = 27) with various capsule K serotypes and sequence types. Phylogenetic relationships were analyzed by Phyloviz 2.0 software using hierarchical clustering based on the genetic distance of sequence types. HM, hypermucoviscous phenotype; SK, serum resistance; SP, siderophore production; 24h-GML, 24-hour lethality in *G mellonella* infection model. Resistance determinants without text meant no other genes carried except *bla*_SHV_ (intrinsic resistance for *K pneumoniae*). **(C)** Antimicrobial resistance rates of hvKp (n = 27) and hgKp-Lv (n = 56). AMP, ampicillin; CXM, cefuroxime; CRO, ceftriaxone; CZO, cefazolin; CTX, cefotaxime; GEN, gentamicin; FEP, cefepime; CAZ, ceftazidime; SXT, trimethoprim-sulfamethoxazole; FOX, cefoxitin; SAM, ampicillin-sulbactam; TZP, piperacillin-tazobactam; AMC, amoxicillin-clavulanic acid; SCF, cefoperazone-sulbactam; FOS, fosfomycin; LVX, levofloxacin; IPM, imipenem; MEM, meropenem; ETP, ertapenem; AMK, amikacin; POL, polymyxin B; TGC, tigecycline. Antimicrobial agents labelled with the same color were grouped into a single antimicrobial category: AMP, Penicillins; CXM/CRO/CZO/CTX/FEP/CAZ/FOX, Cephems; GEN/AMK, Aminoglycosides; SXT, Folate pathway inhibitors; SAM/AMC/SCF/TZP, β lactam combination agents; FOS, Fosfomycins; IPM/ETP/MEM, Carbapenems; LXT, Antipseudomonal fluoroquinolones; TGC, Glycylcyclines; POL, Polymyxins.

Of the 352 K*. pneumoniae* isolates, 83 (23.6%) were hypervirulence genes-positive Kp (hgKp); 35 of the 83 hgKp isolates (42.2%) harbored ≥3 hypervirulence genes, including 25 isolates with five genes that suggested they harbored the full-length pLVPK virulence plasmid (**Figure 1**). Gene *iucA* was present in 67 isolates (80.7%, 67/83), while *prmpA2* was present in 55 isolates (66.3%) (**Figure 2A**). Among the 83 hgKp isolates, 34 (41.0%) exhibited hypermucoviscosity and 46 (55.4%) were resistant to serum killing; 54 isolates (65.1%) and 31 isolates (37.3%) demonstrated virulence comparable to or greater than the positive control NTUH-K2044 in the siderophore production assay and *G. mellonella* infection assay. Overall, 27 isolates (36.1%, 27/83) that showed hypervirulence *in vitro* and *in vivo* were defined as hvKp, whereas 56 isolates (63.9%, 56/83) with hypervirulence genes and generally low-virulence phenotypes were defined as hgKp-low virulence (hgKp-Lv). Compared with hgKp-Lv isolates, hvKp isolates showed hypervirulence characterized by significantly greater rates of hypermucoviscosity, serum killing resistance, and *G. mellonella* lethality (**Table S2**).

### Genetic characteristics and antimicrobial resistance of hypervirulent *K. pneumoniae*

Serotype sequencing showed that the K2 capsule type was present in nine hvKp isolates (33.3%, 9/27), while K1 was present in five hvKp isolates (18.5%) and K57 was present in three hvKp isolates (11.1%) (**Figure 2B**). MLST sequencing revealed 17 distinct sequence types (STs) among the 27 hvKp isolates; ST23 was the most prevalent (14.8%, 4/27), followed by ST25 (11.1%, 3/27). Thus, K1-ST23 (14.8%, 4/27) and K2-ST25 (11.1%, 3/27) were the most common clones among hvKp isolates, followed by K16-ST660, K2-ST86, K57-ST412, and KL47-ST11 (all comprised 7.4%, 2/27). In the hgKp-Lv group, KPC-2-producing KL47-ST11 was the most common clone, and a significant increase was observed in the clone, from 5.3% (1/19) in 2019 to 62.2% (23/37) in 2020 (χ^2^ = 19.5923, *P*<.0001). ERIC-PCR typing identified 24 clusters of hvKp and 31 clusters of hgKp-Lv (**Figure S1**). K1 and K2 capsule types were present in large numbers of hvKp isolates; they were significantly associated with the presence of all five hypervirulence genes (χ^2^ = 7.7489, *P* = 0.0054), which might explain why hvKp isolates had more hypervirulence genes, compared with hgKp-Lv isolates (**Table 1**).

**Table 1:** Comparison of characteristics between hypervirulent *K. pneumoniae* (hvKp) isolates (n = 27) and hypervirulence genes-positive *K. pneumoniae*-low virulence (hgKp-Lv) isolates (n = 56).

	HvKp (n = 27)	HgKp-Lv (n = 56)	*P* Value^a^
**Demographic data**
Age (yrs) (median [IQR]^b^)	3.95 (0.25 - 9.86)	0.12 (0.05 - 0.82)	0.0001^*^ (Z = 3.8443)
Male sex (n [%]^b^)	18 (66.7)	32 (57.1)	0.4062 (χ^2^ = 0.6899)
**Inflammation profile (median [IQR]** ^b^)
White blood cell count (×10^9^/L)	8.03 (5.76 - 13.65)	11.41 (7.86 - 18.72)	0.0447^*^ (Z = -2.0073)
Neutrophil count (%)	51.40 (33.50 - 64.70)	48.70 (31.75 - 65.20)	0.7956 (Z = 0.2590)
C-reactive protein (mg/L)	≤5 (≤5 - 10)	5.5 (≤5 - 14.5)	0.5667 (Z = -0.5729)
Procalcitonin (ng/ml)	0.16 (0.08 - 0.44)	0.29 (0.14 - 0.94)	0.1946 (Z = -1.2971)
**Virulence-associated assays (median [IQR]** ^b^)
Numbers of hypervirulence genes	5 (3.5 - 5)	2 (1.75 - 2)	<.0001^*^ (Z = 5.4348)
Hypermucoviscosity (n [%]^b^)	18 (66.7)	16 (28.6)	0.0009^*^ (χ^2^ = 10.9319)
Grade of serum resistance	5 (5 - 6)	5 (4 - 5)	0.0013^*^ (Z = 3.2113)
Siderophore units (%)^c^	26.0 (6.9 - 36.2)	20.5 (3.6 - 28.4)	0.1258 (Z = 1.5310)
24-hour lethality of Galleria mellonella infection (%)	60 (50 - 80)	15 (10 - 30)	<.0001^*^ (Z = 6.9503)
**Resistance (n [%]** ^b^)
Carbapenemase-producing	3 (11.1)	27 (48.2)	0.0010^*^ (χ^2^ = 10.8656)
MDR	7 (25.9)	37 (66.1)	0.0006^*^ (χ^2^ = 11.7865)
**Infection setting (n [%]** ^b^)
Community acquired	7 (25.9)	5 (8.9)	0.0136^*^ (χ^2^ = 8.5952)
Healthcare associated	10 (37.0)	12 (21.4)	
Hospital acquired	10 (37.0)	39 (69.6)	
**Genotype (n [%]** ^b^)
K1-ST23	4 (14.8)	2 (3.6)	0.0749 (χ^2^ = 3.1718)
K2-ST25	3 (11.1)	3 (5.4)	0.3575 (χ^2^ = 0.8467)
KL47-ST11	2 (7.4)	24 (42.9)	0.0011^*^ (χ^2^ = 10.6416)
K1 and K2	14 (51.9)	6 (10.7)	<.0001^*^ (χ^2^ = 16.8553)
**Outcome (n [%]** ^b^)
Severe pneumonia	6 (22.2)	7 (12.5)	0.4126 (χ^2^ = 0.6714)
30-day crude mortality	1 (3.7)	1 (1.8)	0.5475

a Test statistics are in brackets, P < 0.05 (*).

b Values are shown as median (interquartile range) or No. (%) of cases; Inflammatory indicator data were examined on the same day or within two days of examination for K. pneumoniae pathogens.

c Siderophore units were defined as [(Ar – As)/Ar] × 100 = X%.

Of the 27 hvKp isolates, seven (25.9%) had multi-drug resistance, including three (11.1%) that were CR-hvKp (**Figure 2B**). While 100% of these isolates were intrinsically resistant to ampicillin, most were also resistant to cefuroxime (37.0%), ceftaroline (33.3%), cefmetazole (33.3%), and cefotaxime (33.3%); no hvKp isolates were resistant to polymyxin B or tigecycline (**Figure 2C**). Among the 83 hgKp isolates, 44 were MDR (**Figure S2**). However, compared with the hvKp group, significantly more hgKp-Lv isolates exhibited MDR and carbapenemase-production, with greater resistance to most antimicrobials (**Table 1** and **Figure 2C**). Notably, only two KL47-ST11 hvKp isolates harbored *bla*_KPC-2_, while one K2-ST25 isolate carried *bla*_NDM-1_.

### Genetic and phenotypic screening successfully identified hvKp isolates with severe virulence in mice

**Figure 3:**
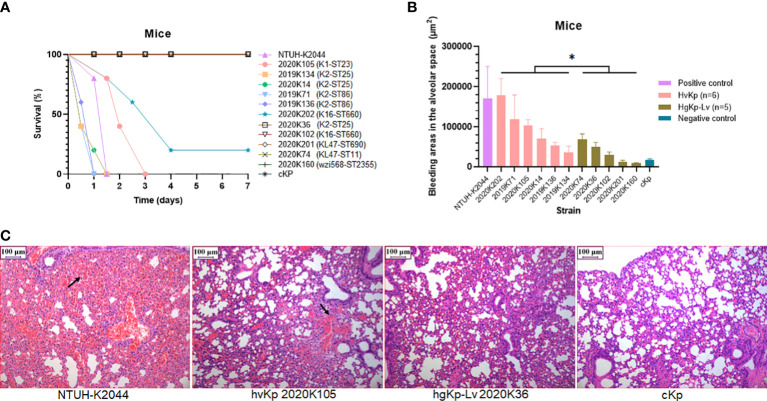
Verification of virulence phenotypes in murine sepsis model using selected hvKp (n = 6) and hgKp-Lv (n = 5) isolates with high-risk clones. **(A)** Mouse survival curves for NTUH-K2044 (hypervirulence control), hvKp isolates (K1-ST23 2020K105, K2-ST25 2019K134, K2-ST25 2020K14, K2-ST86 2019K71, K2-ST86 2019K136, and K16-ST660 2020K202), hgKp-Lv isolates (K2-ST25 2020K36, K16-ST660 2020K102, KL47-ST690 2020K201, KL47-ST11 2020K74, and wzi568-ST2355 2020K160), and cKp (low virulence control). The log-rank (Mantel–Cox) test was performed to assess the indicated curves. A significant difference (χ^2^ = 6.9078, *P* = 0.0086) was observed between the hvKp and hgKp-Lv groups. **(B)** Areas of bleeding in the alveolar space in infected mice. A significant difference (t = 2.33, *P* = 0.0446) was observed between the hvKp and hgKp-Lv groups, according to independent-samples t-test analysis. *P* < 0.05 (*). **(C)** Histological changes in lungs of infected mice. From left to right, images show mouse lungs infected with NTUH-K2044, hvKp 2020K105, hgKp-Lv 2020K36, and cKp, respectively.

To investigate the feasibility of hvKp screening based on four virulence assays, we selected six hvKp and five hgKp-Lv isolates with high-risk clones for confirmation of their *in vivo* virulence potential in a murine sepsis model (**Table S3**), and successful infection in mice was confirmed by consistent strain homology before and after (i.e., after recovery) injection (**Figure S3**). Consistent with our phenotypic classification of hvKp, survival curves significantly differed between hvKp and hgKp-Lv groups (*P* = 0.0086; **Figure 3A**), and 72-hour mortality rates in mice infected with the NTUH-K2044 and cKp strains were 100% and 0%, respectively. Mice infected with five (83.3%) of six hvKp isolates died within 3 days; moreover, mice injected with four (66.7%) of six hvKp isolates died earlier than did mice infected with NTUH-K2044, while mice infected with five (100%) of five hgKp-Lv isolates all survived the monitoring period (7 days).

Notably, areas of bleeding in the alveolar space were significantly greater in hvKp-infected mice than in hgKp-Lv-infected mice (*P* = 0.0446; **Figure 3B, C**). hvKp infection also caused similar damage to other organs (**Figure S4**). Besides, when compared to those infected with hgKp-Lv, mice infected with hvKp had significant increased bacterial burdens reaching 10^8^-10^10^ CFU/g in lung, liver and kidney (**Figure S5**), indicating that hvKp strain is more resistant to clearance by the immune system during systemic infection. These results suggest that a combined assessment approach comprising genetic features and virulence assays (including the *G. mellonella* infection model) can effectively separate hvKp isolates from cKp isolates.

**Figure 4:**
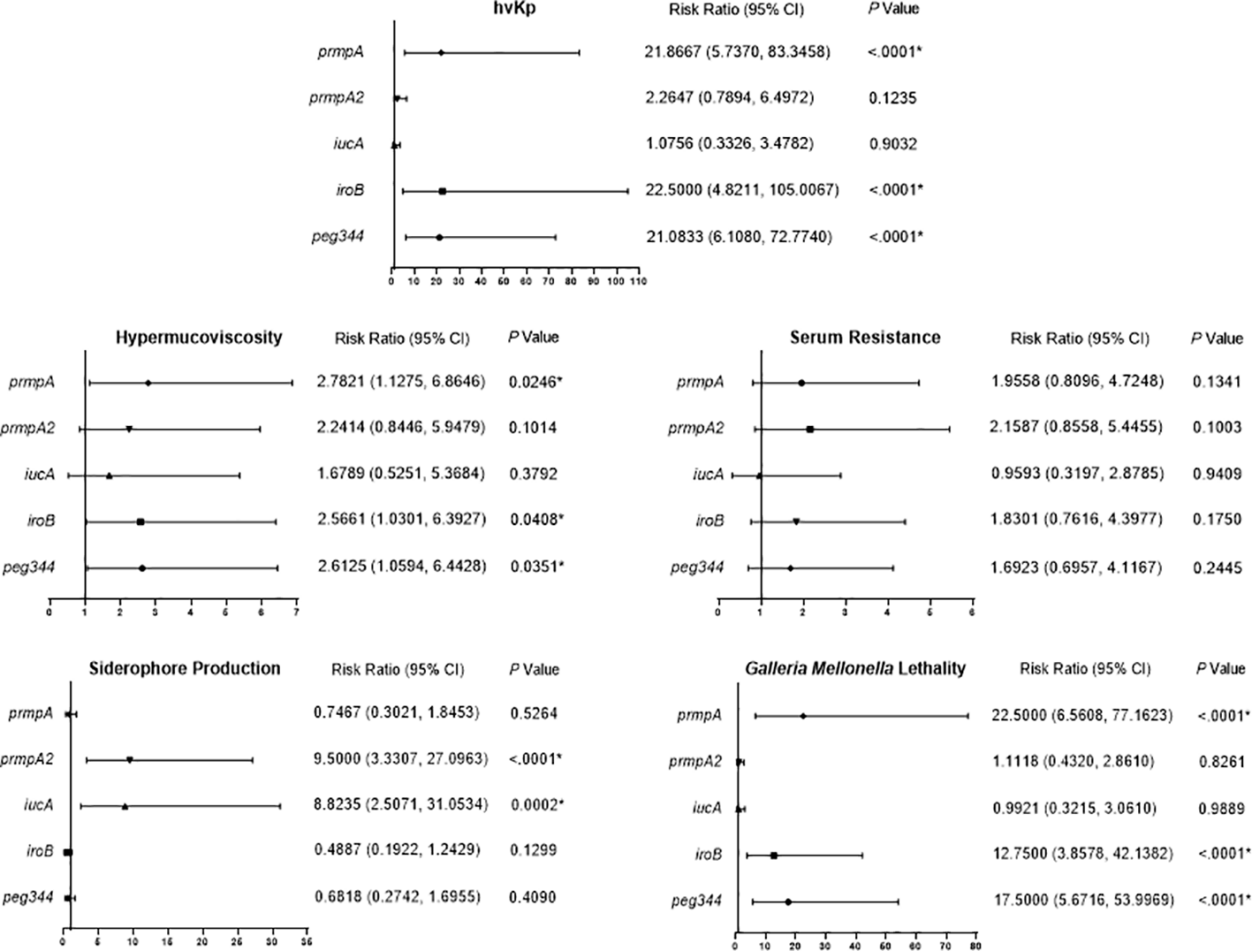
Risk ratio of hypervirulence genes in virulence-associated phenotypes. Risk ratios (95% confidence interval [95% CI]) and *P* values are given for pertinent results. *P* < 0.05 (*).

We analyzed the effects of the five hypervirulence genes on hypervirulence phenotypes; we found that *iucA* and *prmpA2* were significantly associated with excessive siderophore production, while neither gene was associated with serum resistance. However, *prmpA*, *iroB*, and *peg344* were each significantly associated with hypermucoviscosity and *G. mellonella* lethality (**Figure 4**). Furthermore, *K. pneumoniae* harboring *prmpA, iroB*, or *peg344* had a >20-fold greater likelihood of hypervirulence, suggesting that these genes can serve as genetic biomarkers for rapid identification of hvKp in clinical settings.

### Clinical characteristics of hvKp infection

Although patients with hvKp infection did not exhibit a significant sex bias, older children were significantly more likely to develop hvKp infection (**Table S4**). Several critical indicators of acute infection also significantly differed among patients. For example, hepatic function indicator of direct bilirubin (DBIL) and total bilirubin (TBIL), renal function indicator of uric acid, and nutrition indicator total protein, and albumin. However, after multinomial logistic regression with adjustment for age, no significant differences among patients were detected according to infection with hvKp, hgKp-Lv, or cKp.

**Figure 5:**
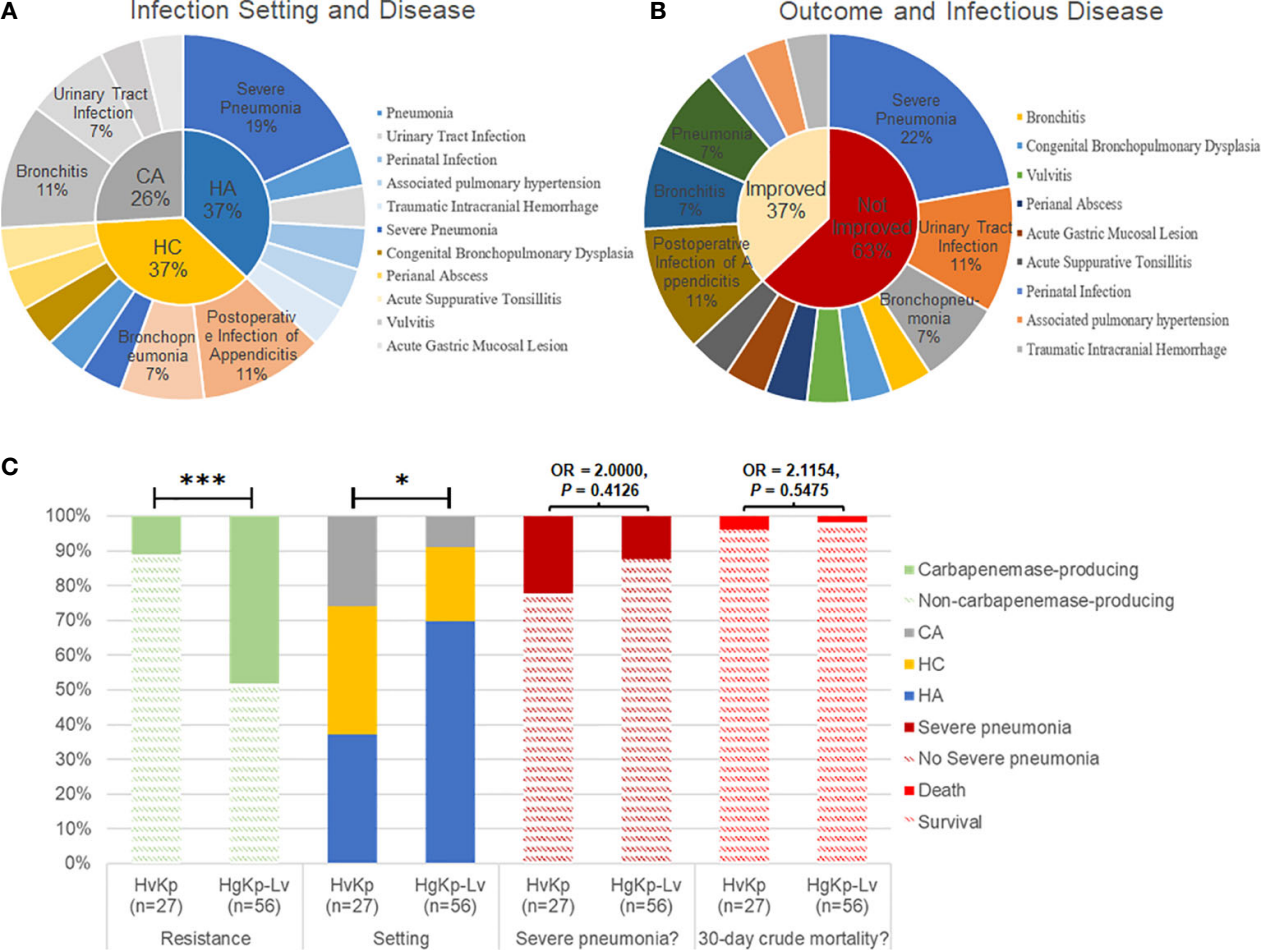
Clinical manifestations of hvKp infection in children. **(A)** Infection setting of hvKp. CA, community-acquired infection; HC, healthcare-associated infection; HA, hospital-acquired infection. **(B)** Outcomes and associated infectious diseases in children with hvKp infection. Outcomes were assessed in terms of prognosis and recurrence during follow-up. **(C)** Comparison of clinical features between hvKp infections and hgKp-Lv infections. Odds ratios (OR) and *P*-values are shown for relevant results. *P* < 0.05 (*) and *P* < 0.001 (***).

Medical records indicated that healthcare-associated and hospital-acquired etiologies each contributed to 37% of hvKp infections. Respiratory infections, including severe pneumonia and bronchitis, occurred in more than half of the patients with hvKp infection (15/27, 55.6%; **Figure 5A, B**). Additionally, most pediatric patients with hvKp infection had adverse outcomes (17/27, 63.0%), mainly because of severe pneumonia (6/27, 22.2%). Compared with patients with hgKp-Lv infection, patients with hvKp infection showed a greater tendency to develop severe pneumoniae (22.2% vs. 12.5%) and death (3.7% vs. 1.8%) (**Figure 5C** and **Table 1**).

## Discussion

To date, there have been few reports of hvKp infection in pediatric patients. In this study, we used the five genotypic biomarkers proposed by Russo et al. (Russo et al., 2018) to identify hypervirulent *K. pneumoniae* isolates in children; we also performed *in vitro* and *in vivo* analyses of virulence phenotypes including hypermucoviscosity, siderophore excessive production, serum killing resistance, *G. mellonella* lethality, and murine sepsis model.

HvKp infection prevalence varies worldwide. Among adults, the prevalence of hvKp was 37.8% (87/230) (Zhang et al., 2016) in China (multicenter, clinical isolates, 2013), 18.6% (26/120) (Harada et al., 2019) in Japan (multicenter, blood isolates only, 2013–2014), 3.7% (17/452) (Parrott et al., 2021) in the USA (New York, invasive isolates only, 2015–2018), 17.8% (157/882) (Lipworth et al., 2021) in the UK (Oxfordshire, blood isolates only, 2008–2018), and 10.9% (267/2432) (Silvester et al., 2022) in lower-middle-income countries (an *in-silico* analysis of Kp from 33 countries available in the NCBI Pathogen Detection system, 2020). The prevalence estimates in these studies relied on at least one genetic biomarker, similar to our use of five virulence genes to identify hgKp. In our study, the estimated prevalence of hgKp in children was 23.6% (83/352), much lower than adults (37.8%, 87/230) (Zhang et al., 2016). Antimicrobial resistance rate was generally low among hvKp isolates, with 25.9% multi-drug resistance and only 11.1% carbapenems resistance, therefore, the prevalence of CR-hgKp was also much lower than adults (23.3%, 30/129 vs. 34.2%, 360/1052) (Zhang et al., 2020). However, in addition to oligonucleotide variants in virulence gene sequences, alterations in the regulatory elements that surround virulence genes can lead to distinct virulence phenotypes in strains with an identical genetic background. Thus, previous gene-based investigations may have overestimated the prevalence of hvKp. For example, *prmpA*/*A2*, regulators of mucoid phenotype genes on the pLVPK-like plasmid, were associated with hypermucoviscosity in this study (χ^2^ = 12.5088, *P* = 0.0004). Strains with *prmpA*-*prmpA2* were mainly enriched in K1/K2 (46.2%, 12/26) and ST23/ST86/ST65 (34.6%, 9/26) hypervirulent clones, causing serious infections as internationally reported (Holt et al., 2015). However, several strains possessing *prmpA*-*prmpA2* did not exhibit hypervirulent phenotypes (26.9%, 7/26; **Table S2**), in consistence with a previous multicenter study (Yang et al., 2022), which might because the genes mutation rendered a loss of function or some unknown genetic traits regulated the hypervirulence expression. Further investigation on *prmpA*-*prmpA2* possessing strains, including phylogenetic analysis with homologous strains available at GeneBank, is required to trace the nature of these factors.

We distinguished between hvKp and hgKp-Lv isolates by using the *G. mellonella* infection model plus positive results in at least one of three other common *in vitro* assays, and hvKp isolates identified in this manner produced significantly more severe disease in mice, compared with hgKp-Lv isolates (**Figure 3**). Nonetheless, the gene-based hvKp screening approach still has its practical significance. *K. pneumoniae* isolates with hypervirulence genes carry a risk of transmission, constituting a substantial public health threat once demonstrating hypervirulence *in vitro* and *in vivo*.

K1 and K2 are traditionally considered as the most common serotype of hvKp (Russo and Marr, 2019), and this trend indeed occurred in children, especially serotype K2 accounting for one third (33.3%, 9/27) in hvKp. Also, we found virulence genes were shifting from traditional K1 and K2 clones to diverse lineages, which resulted in a diversity of clones in hvKp and the predominance of KL47-ST11 in hgKp. However, between K1/K2 and KL47-ST11 strains, a significant difference in virulence *in vitro* and *in vivo* was demonstrated (**Figure 3** and **Table S2**), which was also evidenced by Wang et al. (Wang et al., 2021). Accordingly, KL47-ST11 infection were unable to develop a fatal outbreak (such as the outbreak in Zhejiang (Gu et al., 2018)) in the study, we assumed it might because this type of strains mostly only carried *prmpA2* and *iucA*, without *prmpA*, *iroB*, and *peg344* significantly associated with a hazard ratio of >20 for hypervirulence (**Figure 4**). Anyhow, KL47-ST11 strains pose a substantial threat to children considering they simultaneously harbored hypervirulence genes and carbapenem-resistance genes and had a surge in 2020 due to their high transmissibility. Moreover, to prevent dissemination of hvKp, capsule K1/K2 strains especially high-risk clones K1-ST23/K2-ST25, as well as novel subclone K19-ST660 strains should be concerned.

Sepsis caused by hvKp is often associated with an acute inflammatory response and septic metastases (e.g., liver abscesses). Indeed, in our murine sepsis model, the main organs of hvKp-infected mice all showed greater bleeding tendency and higher bacterial burden, compared with organs from hgKp-infected mice. In contrast, the pathophysiological indicators and mortality rates did not significantly differ among patients according to hvKp or hgKp infection status. This is presumably because many of our hvKp strains were derived from focal infections in non-blood samples, rather than from patients with sepsis. Nevertheless, most hvKp-infected children did not improve after clinical intervention; they tended to exhibit more severe pneumonia and greater mortality, compared with children who had hgKp-Lv infection. Notably, a significant age bias was observed in hvKp infections, such that they more often affected older children (**Table 1**) who may have a longer duration of community residence. This finding may explain the significantly greater risk of community-acquired infection among patients with hvKp isolates than among patients with hgKp-Lv isolates.

In conclusion, gene-based screening followed by phenotype validation was effective for identification of hvKp. K1 and K2 capsules remained the most common hvKp genotypes. However, we found novel hypervirulent subclones of K2-ST25 and K19-ST660. Hypervirulence genes are spreading to a wide range of capsular types. hvKp infections in children were mostly nosocomial or healthcare-associated and commonly involved severe pneumonia; medical institutions should carefully implement nosocomial infection control and surveillance, along with timely interventions. Although our findings were derived from a single center with a limited number of samples, they suggest dramatic spread of hvKp in children and emphasize the urgent need for further epidemiological studies of hvKp.

## Data availability statement

The original contributions presented in the study are included in the article/**Supplementary Material**. Further inquiries can be directed to the corresponding authors.

## Ethics statement

This retrospective study was approved by the Shanghai Children's Hospital Institutional Review Board. The animal study was reviewed and approved by Shanghai Children’s Hospital Ethics Committee.

## Author contributions

Conceptualization: QD, DH, and HZ; data curation: QD; Formal analysis: QD; funding acquisition: DH and HZ; methodology: QD, FP, CW, FY, YS, WL, ZL, and PH; project administration: DH and HZ; supervision: HZ; validation: QD, FP, DH, and HZ; writing (original draft): QD; writing (review and editing): FP, DH, and HZ. All authors contributed to the article and approved the submitted version.

## Funding

This work was supported by Shanghai Municipal Key Clinical Specialty (shslczdzk06902), Natural Science Foundation of Shanghai (21ZR1452900).

## Acknowledgments

We thank Bingjie Wang from Shanghai Pulmonary Hospital, Dongxing Tian from Huashan Hospital of Fudan University and Qi Xu from Ruijin Hospital for assistance with animal experiments and siderophores production assays.

## Conflict of interest

The authors declare that the research was conducted in the absence of any commercial or financial relationships that could be construed as a potential conflict of interest.

## Publisher’s note

All claims expressed in this article are solely those of the authors and do not necessarily represent those of their affiliated organizations, or those of the publisher, the editors and the reviewers. Any product that may be evaluated in this article, or claim that may be made by its manufacturer, is not guaranteed or endorsed by the publisher.
