# Supplementary material for: Nosocomial dissemination of hypervirulent Klebsiella pneumoniae with high-risk clones among children in Shanghai
Source: Front Cell Infect Microbiol. 2022 Aug 29;12:984180. doi: 10.3389/fcimb.2022.984180 (PMC9464974; doi:10.3389/fcimb.2022.984180)
Supplement: Supplementary file 1 [file Table_1.docx]

**Supplementary**

Table S1. Primers in this study.

| Gene | Primers | Length (bp) | Annealing temperature (°C) | References |
| --- | --- | --- | --- | --- |
| Hypervirulence genes (mutiplex PCR) | | | | |
| *rmpA-*F | ATGTGGCTTGACGTTTCGGGGG | 160 | 60 | [1] |
| *rmpA-*R | GCCGTGGATAATGGTTTACAATTCGGC |  |  |  |
| *rmpA2*-F | GGATGTGGCTTGACATTTCGGGGG | 227 |  |  |
| *rmpA2-*R | TTCATGGATGCCCTCCCTCCTG |  |  |  |
| *HI1B-F1* | TCGCTACTGCGATTGGGGGTCT | 351 |  |  |
| *HI1B-R1* | GAAATGGGTGTGCTGGAGCCGT |  |  |  |
| *iroN-F1* | CCGCAAAGAGACGAACCGCCTT | 546 |  |  |
| *iroN-R1* | CGGGCAATCCCCGCTTTGACTT |  |  |  |
| *iutA-F1* | AATCACCTGGGGGCTGGATGCT | 683 |  |  |
| *iutA-R1* | CCGCACCTTCCACGCCGTAAAT |  |  |  |
| Hypervirulence genes (monoplex PCR) | | | | |
| *prmpA*-F | TACATATGAAGGAGTAGTTAAT | 505 | 42 | [2] |
| *prmpA*-R | GAGCCATCTTTCATCAAC |  |  |  |
| *prmpA2*-F | TGTGCAATAAGGATGTTACATTAGT | 607 | 57 |  |
| *prmpA2*-R | TTTGATGTGCACCATTTTTCA |  |  |  |
| *iucA-*F | AATCAATGGCTATTCCCGCTG | 239 | 52 |  |
| *iucA-*R | CGCTTCACTTCTTTCACTGACAGG |  |  |  |
| *iroB-*F | CAAAAAAGCAGCAGAGGC | 585 | 52 |  |
| *iroB-*R | TCACTGGCGGAATCCAACAC |  |  |  |
| *peg344-*F | CTTGAAACTATCCCTCCAGTC | 508 | 52 |  |
| *peg344-*R | CCAGCGAAAGAATAACCCC |  |  |  |
| *rmpA*-F | GCAGTTAACTGGACTACCTCTG | 322 | 56 | [3] |
| *rmpA*-R | GTTTACAATTCGGCTAACATTTTTCTTTAAG |  |  |  |
| *aerobactin*-F | GCATAGGCGGATACGAACAT | 556 | 50 | [4] |
| *aerobactin*-R | CACAGGGCAATTGCTTACCT |  |  |  |
| Resistance genes (Carbapenemases) | | | | |
| bla*_NDM_-*F | ATGGAATTGCCCAATATTATGC | 813 | 55 | [5] |
| bla*_NDM_-*R | TCAGCGCAGCTTGTCGGC |  |  |  |
| *bla_KPC_*-F | AGGACTTTGGCGGCTCCAT | 720 | 55 |  |
| *bla_KPC_*-R | TCCCTCGAGCGCGAGTCTA |  |  |  |
| *bla_OXA-48_*-F | GCGTGGTTAAGGATGAACAC | 438 | 52 |  |
| *bla_OXA-48_*-R | CATCAAGTTCAACCCAACCG |  |  |  |
| *bla_IMP_*-F | GGAATAGAGTGGCTTAAYTCTC | 232 | 52 |  |
| *bla_IMP_*-R | GGTTTAAYAAAACAACCACC |  |  |  |
| *bla_AIM_*-F | CTGAAGGTGTACGGAAACAC | 322 | 52 |  |
| *bla_AIM_*-R | GTTCGGCCACCTCGAATTG |  |  |  |
| *bla_VIM_*-F | GATGGTGTTTGGTCGCATA | 390 | 52 |  |
| *bla_VIM_*-R | CGAATGCGCAGCACCAG |  |  |  |
| *bla_GIM_*-F | TCGACACACCTTGGTCTGAA | 477 | 52 |  |
| *bla_GIM_*-R | AACTTCCAACTTTGCCATGC |  |  |  |
| *bla_SIM_*-F | TACAAGGGATTCGGCATCG | 570 | 52 |  |
| *bla_SIM_-R* | AATGGCCTGTTCCCATGTG |  |  |  |
| Resistance genes (ESBLs) | | | | |
| *bla_TEM_*-F | TCCGCTCATGAGACAATAACC | 296 | 52 | [6] |
| *bla_TEM_*-R | ATAATACCGCACCACATAGCAG |  |  |  |
| *bla_SHV_*-F | TACCATGAGCGATAACAGCG | 450 |  |  |
| *bla_SHV_*-R | GATTTGCTGATTTCGCTCGG |  |  |  |
| *bla_CTX-M_-F* | CAAAGAGAGTGCAACGGATG | 205 |  | [7] |
| *bla_CTX-M_-R* | ATTGGAAAGCGTTCATCACC |  |  |  |
| Resistance genes (AmpC) | | | | |
| *MOX-F* | GCTGCTCAAGGAGCACAGGAT | 520 | 64 | [8] |
| *MOX-R* | CACATTGACATAGGTGTGGTGC |  |  |  |
| *CIT-F* | TGGCCAGAACTGACAGGCAAA | 462 |  |  |
| *CIT-R* | TTTCTCCTGAACGTGGCTGGC |  |  |  |
| *DHA-F* | AACTTTCACAGGTGTGCTGGGT | 405 |  |  |
| *DHA-R* | CCGTACGCATACTGGCTTTGC |  |  |  |
| *ACC-F* | AACAGCCTCAGCAGCCGGTTA | 346 |  |  |
| *ACC-R* | TTCGCCGCAATCATCCCTAGC |  |  |  |
| *EBC-F* | TCGGTAAAGCCGATGTTGCGG | 302 |  |  |
| *EBC-R* | CTTCCACTGCGGCTGCCAGTT |  |  |  |
| *FOX-F* | AACATGGGGTATCAGGGAGATG | 190 |  |  |
| *FOX-R* | CAAAGCGCGTAACCGGATTGG |  |  |  |
| Capsule serotype | | | | |
| *wzi-F* | GTGCCGCGAGCGCTTTCTATCTTGGTATTCC | | 55 | [9] |
| *wzi-R* | GAGAGCCACTGGTTCCAGAATTTGACCGC | |  |  |
| K1-F | GGTGCTCTTTACATCATTGC | 1283 | 50 | [10] |
| K1-R | GCAATGGCCATTTGCGTTAG |  |  |  |
| K2-F | GGATTATGACAGCCTCTCCT | 908 | 52 |  |
| K2-R | CGACTTGGTCCCAACAGTTT |  |  |  |
| K5-F | CAGGGAACTCCTACGCAGATTT | 1036 | 54 |  |
| K5-R | GGGTGATAAGGTATAGCTGACAC |  |  |  |
| K20-F | CGGTGCTACAGTGCATCATT | 741 | 52 |  |
| K20-R | GTTATACGATGCTCAGTCGC |  |  |  |
| K54-F | GTTACCTCAGAGCGTTGCAT | 974 | 53 |  |
| K54-R | CGGACTTAATAGCGAGCAAAG |  |  |  |
| K57-F | CTCAGGGCTAGAAGTGTCAT | 1037 | 52 |  |
| K57-R | CACTAACCCAGAAAGTCGAG |  |  |  |
| KL47-F | GGACGCACAGTTTCCCAATTCGC | 392 | 60 | [1] |
| KL47-R | GCCCACATGAACCCACTTGGCA |  |  |  |
| KL64-F | TCAGTTCCGACCCTGATGCAGGTA | 268 |  |  |
| KL64--R | GCCAGAGCAACTATCATCCAAAGCCA |  |  |  |
| Plasmid replicons | | | | |
| HI1 FW | GGAGCGATGGATTACTTCAGTAC | 471 | 60 | [11] |
| HI1 RV | TGCCGTTTCACCTCGTGAGTA |  |  |  |
| HI2 FW | TTTCTCCTGAGTCACCTGTTAACAC | 644 |  |  |
| HI2 RV | GGCTCACTACCGTTGTCATCCT |  |  |  |
| I1 FW | CGAAAGCCGGACGGCAGAA | 139 |  |  |
| I1 RV | TCGTCGTTCCGCCAAGTTCGT |  |  |  |
| XFW | AACCTTAGAGGCTATTTAAGTTGCTGAT | 376 | 60 |  |
| XRV | TGAGAGTCAATTTTTATCTCATGTTTTAGC |  |  |  |
| L/M FW | GGATGAAAACTATCAGCATCTGAAG | 785 |  |  |
| L/M RV | CTGCAGGGGCGATTCTTTAGG |  |  |  |
| NFW | GTCTAACGAGCTTACCGAAG | 559 |  |  |
| NRV | GTTTCAACTCTGCCAAGTTC |  |  |  |
| FIA FW | CCATGCTGGTTCTAGAGAAGGTG | 462 | 60 |  |
| FIA RV | GTATATCCTTACTGGCTTCCGCAG |  |  |  |
| FIB FW | GGAGTTCTGACACACGATTTTCTG | 702 |  |  |
| FIB RV | CTCCCGTCGCTTCAGGGCATT |  |  |  |
| WFW | CCTAAGAACAACAAAGCCCCCG | 242 |  |  |
| WRV | GGTGCGCGGCATAGAACCGT |  |  |  |
| YFW | AATTCAAACAACACTGTGCAGCCTG | 765 | 60 |  |
| YRV | GCGAGAATGGACGATTACAAAACTTT |  |  |  |
| PFW | CTATGGCCCTGCAAACGCGCCAGAAA | 534 |  |  |
| PRV | TCACGCGCCAGGGCGCAGCC |  |  |  |
| FIC FW | GTGAACTGGCAGATGAGGAAGG | 262 |  |  |
| FIC RV | TTCTCCTCGTCGCCAAACTAGAT |  |  |  |
| A/C FW | GAGAACCAAAGACAAAGACCTGGA | 465 | 60 |  |
| A/C RV | ACGACAAACCTGAATTGCCTCCTT |  |  |  |
| TFW | TTGGCCTGTTTGTGCCTAAACCAT | 750 |  |  |
| TRV | CGTTGATTACACTTAGCTTTGGAC |  |  |  |
| FIIS FW | CTGTCGTAAGCTGATGGC | 270 |  |  |
| FIIS RV | CTCTGCCACAAACTTCAGC |  |  |  |
| FrepBFW | TGATCGTTTAAGGAATTTTG | 270 | 50 |  |
| FrepB RV | GAAGATCAGTCACACCATCC |  |  |  |
| K/B FW | GCGGTCCGGAAAGCCAGAAAAC | 160 | 50 |  |
| KRV | TCTTTCACGAGCCCGCCAAA |  |  |  |
| B/O RV | TCTGCGTTCCGCCAAGTTCGA | 159 | 50 |  |
| ERIC-PCR | | | | |
| ERIC1 | ATGTAAGCTCCTGGGGATTCAC | | 49 | [12] |
| ERIC2 | AAGTAAGTGAC TGGGGTGAGCG | |  |  |
| MLST | | | | |
| *rpoB-F* | GTTTTCCCAGTCACGACGTTGTAGGCGAAATGGCWGAGAACCA | | 50 | [13] |
| *rpoB*-R | TTGTGAGCGGATAACAATTTCGAGTCTTCGAAGTTGTAACC | |  |  |
| *gapA-F* | GTTTTCCCAGTCACGACGTTGTATGAAATATGACTCCACTCACGG | | 50 |  |
| *gapA-F* | TTGTGAGCGGATAACAATTTCCTTCAGAAGCGGCTTTGATGGCTT | |  |  |
| *mdh-F* | GTTTTCCCAGTCACGACGTTGTACCCAACTCGCTTCAGGTTCAG | | 50 |  |
| *mdh-R* | TTGTGAGCGGATAACAATTTCCCGTTTTTCCCCAGCAGCAG | |  |  |
| *pgi-F* | GTTTTCCCAGTCACGACGTTGTAGAGAAAAACCTGCCTGTACTGCTGGC | | 50 |  |
| *pgi-R* | TTGTGAGCGGATAACAATTTCCGCGCCACGCTTTATAGCGGTTAAT | |  |  |
| *phoE-F* | GTTTTCCCAGTCACGACGTTGTAACCTACCGCAACACCGACTTCTTCGG | | 50 |  |
| *phoE-R* | TTGTGAGCGGATAACAATTTCTGATCAGAACTGGTAGGTGAT | |  |  |
| *infB-F* | GTTTTCCCAGTCACGACGTTGTACTCGCTGCTGGACTATATTCG | | 50 |  |
| *infB-R* | TTGTGAGCGGATAACAATTTC CGCTTTCAGCTCAAGAACTTC | |  |  |
| *tonB-F* | GTTTTCCCAGTCACGACGTTGTACTTTATACCTCGGTACATCAGGTT | | 50 |  |
| *tonB-R* | TTGTGAGCGGATAACAATTTCATTCGCCGGCTGRGCRGAGAG | |  |  |

[1] Yu F, Lv J, Niu S, et al. Multiplex PCR Analysis for Rapid Detection of Klebsiella pneumoniae Carbapenem-Resistant (Sequence Type 258 [ST258] and ST11) and Hypervirulent (ST23, ST65, ST86, and ST375) Strains. J Clin Microbiol. 2018 Aug 27;56(9):e00731-18.

[2] Russo TA, Olson R, Fang CT, et al. Identification of Biomarkers for Differentiation of Hypervirulent Klebsiella pneumoniae from Classical K. pneumoniae. J Clin Microbiol. 2018 Aug 27;56(9):e00776-18.

[3] Fang CT, Lai SY, Yi WC, et al. Klebsiella pneumoniae genotype K1: an emerging pathogen that causes septic ocular or central nervous system complications from pyogenic liver abscess. Clin Infect Dis. 2007 Aug 1;45(3):284-93.

[4] Zhang Y, Zhao C, Wang Q, et al. High Prevalence of Hypervirulent Klebsiella pneumoniae Infection in China: Geographic Distribution, Clinical Characteristics, and Antimicrobial Resistance. Antimicrob Agents Chemother. 2016;60(10):6115-6120. Published 2016 Sep 23.

[5] Poirel L, Walsh T R, Cuvillier V, et al. Multiplex PCR for detection of acquired carbapenemase genes. Diagn Microbiol Infect Dis. 2011 May;70(1):119-23.

[6] Doosti A, Pourabbas M, Arshi A, et al. TEM and SHV Genes in Klebsiella pneumoniae Isolated from Cockroaches and Their Antimicrobial Resistance Pattern. Osong Public Health Res Perspect. 2015 Feb;6(1):3-8.

[7] Dallenne C, Da Costa A, Decre D, et al. Development of a set of multiplex PCR assays for the detection of genes encoding important beta-lactamases in Enterobacteriaceae. J Antimicrob Chemother. 2010 Mar;65(3):490-5.

[8] Perez-Perez F J, Hanson N D. Detection of plasmid-mediated AmpC beta-lactamase genes in clinical isolates by using multiplex PCR. J Clin Microbiol. 2002 Jun;40(6):2153-62.

[9] Brisse S, Passet V, Haugaard A B, et al. wzi Gene sequencing, a rapid method for determination of capsular type for Klebsiella strains. J Clin Microbiol. 2013 Dec;51(12):4073-8.

[10] Li W, Sun G, Yu Y, et al. Increasing occurrence of antimicrobial-resistant hypervirulent (hypermucoviscous) Klebsiella pneumoniae isolates in China. Clin Infect Dis. 2014 Jan;58(2):225-32.

[11] Carattoli A, Bertini A, Villa L, et al. Identification of plasmids by PCR-based replicon typing. J Microbiol Methods. 2005 Dec;63(3):219-28.

[12] Versalovic J, Koeuth T, Lupski JR. Distribution of repetitive DNA sequences in eubacteria and application to fingerprinting of bacterial genomes. Nucleic Acids Res. 1991 Dec 25;19(24):6823-31.

[13] Diancourt L, Passet V, Verhoef J, et al. Multilocus sequence typing of Klebsiella pneumoniae nosocomial isolates. J Clin Microbiol. 2005 Aug;43(8):4178-82.

Table S2. Epidemiological characteristics of hypervirulence genes-positive *K. pneumoniae* (hgKp) (n=83).

|  | K type-ST^a^ (n) | Virulence genes (n) | HM^b^ (n) | HSK^b^ (n) | ESP^b^ (n) | HGML^b^ (n) | Resistance determinants (n) | Antimicrobial resistance^c^ (R [n]) | Plasmid replicons (n) |
| --- | --- | --- | --- | --- | --- | --- | --- | --- | --- |
| hvKp (n=27) | K1-ST23 (4) | *prmpA-prmpA2*-*iucA*-*iroB*-*peg344* (4) | 2 | 3 | 4 | 4 | *bla*_SHV_ (3), *bla*_SHV_-*bla*_CTX_-*bla*_DHA_ (1) | GEN (1), AMP (4), CRO (1), CZO (1), CXM (1), CTX (1), FEP (1), CAZ (1), LVX (1) | IncF-IncHI1B (3), IncF-IncFIB-IncHI1B (1) |
|  | K1-ST82 (1) | *prmpA*-*iucA* (1) | 1 | 1 | 1 | 1 | *bla*_SHV_-*bla*_TEM_ (1) | GEN (1), AMP (1), CRO (1), CZO (1), CXM (1), CTX (1) | IncF-IncFII (1) |
|  | K2-ST25 (3) | *prmpA-iroB*-*peg344* (3) | 2 | 3 | 0 | 3 | *bla*_SHV_ (2), *bla*_NDM-1_-*bla*_SHV_-*bla*_CTX_-*bla*_DHA_-*bla*_EBC_ (1) | GEN (1), AMP (3), CRO (1), CZO (1), CXM (2), CTX (1), FEP (1), CAZ (1), FOX (2), SXT (2), SAM (1), TZP (1), AMC (1), SCF (1), IPM (1), MEM (1), ETP (1) | IncF (1), IncF-IncFIB-IncFII (1), IncF-IncFIB-IncHI1B (1) |
|  | K2-ST65 (1) | *prmpA-prmpA2*-*iucA*-*iroB* (1) | 1 | 1 | 1 | 1 | *bla*_SHV_ (1) | AMP (1) | IncF-IncFIB-IncHI1B (1) |
|  | K2-ST86 (2) | *prmpA-prmpA2*-*iucA*-*iroB*-*peg344* (2) | 2 | 2 | 2 | 2 | *bla*_SHV_ (2) | AMP (2) | IncFIB-IncHI1B (1), IncF-IncFIB-IncHI1B (1) |
|  | K2-ST375 (1) | *prmpA-prmpA2*-*iucA*-*iroB*-*peg344* (1) | 1 | 1 | 1 | 1 | *bla*_SHV_ (1) | AMP (1) | IncFIB-IncHI1B (1) |
|  | K2-ST570 (1) | *prmpA-prmpA2*-*iucA*-*iroB*-*peg344* (1) | 1 | 1 | 1 | 1 | *bla*_SHV_ (1) | AMP (1) | IncF-IncHI1B (1) |
|  | K2-ST680 (1) | *prmpA-prmpA2*-*iucA*-*iroB*-*peg344* (1) | 1 | 1 | 1 | 1 | *bla*_SHV_-*bla*_TEM_-*bla*_CTX_-*bla*_EBC_ (1) | AMP (1), CRO (1), CZO (1), CXM (1), CTX (1), FEP (1), CAZ (1), SXT (1), SAM (1), TZP (1), AMC (1), SCF (1) | IncF (1) |
|  | K16-ST660 (2) | *prmpA-prmpA2*-*iucA*-*iroB*-*peg344* (2) | 2 | 0 | 2 | 2 | *bla*_SHV_ (2) | AMP (2) | IncF-IncFII-IncHI1B (2) |
|  | K20-ST420 (1) | *prmpA-prmpA2*-*iucA*-*iroB*-*peg344* (1) | 1 | 1 | 1 | 1 | *bla*_SHV_ (1) | AMP (1) | IncF-IncFIB-IncHI1B (1) |
|  | K23-ST280 (1) | *prmpA2*-*iroB* (1) | 0 | 0 | 1 | 1 | *bla*_SHV_-*bla*_CTX_ (1) | GEN (1), AMP (1), CRO (1), CZO (1), CXM (2), CTX (1) | IncFIB-IncFII (1) |
|  | K57-ST218 (1) | *prmpA-prmpA2*-*iucA*-*iroB*-*peg344* (1) | 1 | 1 | 1 | 1 | *bla*_SHV_ (1) | AMP (1), CRO (1), CXM (1), CTX (1), SXT (1) | IncFIB-IncFII (1) |
|  | K57-ST347 (1) | *prmpA-prmpA2*-*iucA*-*iroB*-*peg344* (1) | 1 | 1 | 0 | 1 | *bla*_SHV_ (1) | AMP (1) | IncF (1) |
|  | K57-ST412 (2) | *prmpA-prmpA2*-*iucA*-*iroB*-*peg344* (2) | 0 | 1 | 1 | 2 | *bla*_SHV_ (2) | AMP (2) | IncF-IncFIB (1) |
|  | KL47-ST11 (2) | *prmpA2-iucA-iroB-peg344* (1), *prmpA-prmpA2*-*iucA*-*iroB*-*peg344* (1) | 1 | 2 | 2 | 2 | *bla*_SHV_ (1), *bla*_KPC-2_-*bla*_SHV_-*bla*_CTX_ (1) | FOS (2), GEN (2), AMK (2), AMP (2), CRO (2), CZO (2), CXM (2), CTX (2), FEP (2), CAZ (2), FOX (2), LVX (2), SXT (1), SAM (2), TZP (2), AMC (2), SCF (2), IPM (2), MEM (2), ETP (2) | IncF-IncFII (1), IncF-IncFIB-IncFII-IncHI1B (1) |
|  | KL47-ST441 (1) | *prmpA-iroB*-*peg344* (1) | 0 | 0 | 1 | 1 | *bla*_SHV_ (1) | AMP (1), CRO (1), CZO (1), CXM (1), CTX (1) | IncF-IncFII (1) |
|  | KL107-ST4316 (1) | *prmpA-prmpA2*-*iucA*-*iroB*-*peg344* (1) | 1 | 1 | 0 | 1 | *bla*_SHV_ (1) | AMP (1) | IncF (1) |
|  | KL127-ST4316 (1) | *iroB* (1) | 0 | 1 | 1 | 1 | *bla*_SHV_ (1) | AMP (1) | IncF (1) |
| hgKp-Lv (n=56) | K1-ST23 (2) | *prmpA-prmpA2*-*iucA*-*iroB*-*peg344* (2) | 1 | 1 | 1 | 0 | *bla*_SHV_ (2) | AMP (2) | IncFIB (1), IncF-IncFII (1) |
|  | K2-ST25 (3) | *prmpA-iroB* (1), *prmpA*-*iucA*-*iroB*-*peg344* (2) | 0 | 0 | 1 | 2 | *bla*_SHV_ (1), *bla*_SHV_-*bla*_TEM_-*bla*_CTX_ (2) | FOS (1), AMP (3), CRO (2), CZO (2), CXM (2), CTX (2), FEP (2), CAZ (2), LVX (2), SXT (3), SAM (2), TZP (2), AMC (2), SCF (2) | IncF (1), IncFIB (1), IncHI1B (1) |
|  | K2-ST584 (1) | *iucA* (1) | 0 | 0 | 1 | 0 | *bla*_SHV_ (1) | AMP (1), AMC (1) | IncF-IncFIB-IncFII-IncHI1B (1) |
|  | K9-ST1440 (1) | *iroB*-*peg344* (1) | 0 | 0 | 1 | 0 | *bla*_SHV_-*bla*_TEM_-*bla*_CTX_-*bla*_DHA_-*bla*_EBC_-*bla*_FOX_ (1) | FOS (1), GEN (1), AMK (1), AMP (1), CRO (1), CZO (1), CXM (1), CTX (1), FEP (1), CAZ (1), FOX (1), LVX (1), SXT (1), SAM (1), TZP (1), AMC (1), SCF (1) | IncF (1) |
|  | K16-ST660 (1) | *prmpA-prmpA2*-*iucA*-*iroB*-*peg344* (1) | 1 | 0 | 0 | 0 | *bla*_SHV_ (1) | AMP (1) | IncF-IncFIB-IncFII (1) |
|  | K16-ST37 (1) | *prmpA-prmpA2*-*iucA*-*iroB*-*peg344* (1) | 1 | 0 | 0 | 0 | *bla*_SHV_ (1) | AMP (1) | IncFIB (1) |
|  | K27-ST335 (1) | *iroB*-*peg344* (1) | 0 | 0 | 0 | 0 | *bla*_SHV_ (1) | AMP (1) | IncF-IncFIB (1) |
|  | K27-ST661 (1) | *iucA* (1) | 1 | 1 | 0 | 0 | *bla*_SHV_-*bla*_TEM_-*bla*_CTX_-*bla*_EBC_ (1) | AMP (1), CRO (1), CZO (1), CXM (1), CTX (1), FEP (1), CAZ (1), SXT (1), SAM (1), TZP (1), AMC (1), SCF (1) | IncF (1) |
|  | K30-ST198 (1) | *prmpA-iucA*-*iroB*-*peg344* (1) | 0 | 1 | 0 | 0 | *bla*_SHV_-*bla*_TEM_-*bla*_CTX_ (1) | GEN (1), AMP (1), CRO (1), CZO (1), CXM (1), CTX (1), FEP (1), CAZ (1), SXT (1), TGC (1), SAM (1), SCF (1) | IncFIB (1) |
|  | K54-ST2239 (1) | *iroB* (1) | 0 | 1 | 0 | 0 | *bla*_SHV_ (1) | AMP (1) | IncF-IncFIB-IncFII (1) |
|  | K81-ST831 (2) | *prmpA2* (1)*, iroB* (1) | 2 | 2 | 0 | 0 | *bla*_SHV_ (1), *bla*_SHV_-*bla*_CTX_ (1) | AMP (2), CRO (1), CZO (1), CXM (1), CTX (1), CAZ (1), FOX (1), SXT (1), SAM (1), AMC (1), SCF (1) | IncF (1), IncF-IncFIB-IncFII (1) |
|  | KL47-ST11 (24) | *prmpA2*-*iucA* (23), *prmpA*-*prmpA2*-*iucA*-*iroB*-*peg34*4 (1) | 6 | 14 | 24 | 0 | *bla*_KPC-2_-*bla*_SHV_-*bla*_CTX_ (19), *bla*_KPC-2_-*bla*_SHV_-*bla*_TEM_-*bla*_CTX_ (2), *bla*_KPC-2_-*bla*_SHV_ (1), *bla*_KPC-2_-*bla*_SHV_-*bla*_CTX_-*bla*_ACC_ (1), *bla*_KPC-2_-*bla*_SHV_-*bla*_TEM_-*bla*_CTX_-*bla*_DHA_-*bla*_EBC_ (1) | FOS (18), GEN (23), AMK (23), AMP (24), CRO (24), CZO (24), CXM (24), CTX (24), FEP (24), CAZ (24), FOX (24), LVX (18), SXT (1), SAM (24), TZP (24), AMC (24), SCF (24), IPM (24), MEM (24), ETP (24) | IncF-IncFII-IncHI1B (18), IncF-IncFIB-IncFII-IncHI1B (2), IncF-IncFII (2), IncF-IncFIB-IncFII (1), IncF-IncHI1B (1) |
|  | KL47-ST15 (1) | *prmpA* (1) | 0 | 0 | 0 | 1 | *bla*_SHV_-*bla*_TEM_-*bla*_CTX_ (1) | GEN (1), AMP (1), CRO (1), CZO (1), CXM (1), CTX (1), FEP (1), CAZ (1), FOX (1), SAM (1), TZP (1), AMC (1), SCF (1) | IncF-IncFII (1) |
|  | KL47-ST36 (1) | *iroB* (1) | 0 | 1 | 0 | 0 | *bla*_SHV_ (1) | AMP (1), LVX (1) | IncF-IncFIB (1) |
|  | KL47-ST76 (1) | *prmpA2*-*iucA* (1) | 1 | 0 | 1 | 0 | *bla*_KPC-2_-*bla*_SHV_-*bla*_CTX_ (1) | AMP (1), CRO (1), CZO (1), CXM (1), CTX (1), FEP (1), CAZ (1), SXT (1), TGC (1), SAM (1), TZP (1), AMC (1), SCF (1), IPM (1), MEM (1), ETP (1) | IncF-IncFIB (1) |
|  | KL47-ST163 (1) | *prmpA* (1) | 0 | 0 | 0 | 0 | *bla*_SHV_ (1) | AMP (1) | IncF-IncHI1B (1) |
|  | KL47-ST298 (1) | *prmpA2*-*iucA* (1) | 0 | 0 | 0 | 0 | *bla*_SHV_ (1) | AMP (1) | IncF-IncFII-IncHI1B (1) |
|  | KL47-ST690 (1) | *prmpA2*-*iucA*-*iroB* (1) | 0 | 0 | 1 | 0 | *bla*_KPC-2_-*bla*_NDM-1_-*bla*_SHV_-*bla*_CTX_ (1) | GEN (1), AMK (1), AMP (1), CRO (1), CZO (1), CXM (1), CTX (1), FEP (1), CAZ (1), FOX (1), SAM (1), TZP (1), AMC (1), SCF (1), IPM (1), MEM (1), ETP (1) | IncF-IncFIB (1) |
|  | KL47-ST3483 (1) | *prmpA-prmpA2*-*iucA*-*iroB*-*peg344* (1) | 0 | 0 | 1 | 0 | *bla*_SHV_ (1) | AMP (1) | IncF-IncFIB-IncFII-IncHI1B (1) |
|  | KL64-ST1869 (1) | *prmpA-prmpA2*-*iucA*-*iroB*-*peg344* (1) | 0 | 1 | 0 | 0 | *bla*_KPC-2_-*bla*_SHV_-*bla*_TEM_-*bla*_CTX_ (1) | FOS (1), GEN (1), AMK (1), AMP (1), CRO (1), CZO (1), CXM (1), CTX (1), FEP (1), CAZ (1), FOX (1), LVX (1), SXT (1), SAM (1), TZP (1), AMC (1), SCF (1), IPM (1), MEM (1), ETP (1) | IncF-IncFIB-IncFII (1) |
|  | KL64-ST3712 (1) | *iucA* (1) | 0 | 0 | 0 | 1 | *bla*_SHV_ (1) | AMP (1) | IncF-IncFII (1) |
|  | KL134-ST18 (1) | *iucA* (1) | 0 | 0 | 0 | 0 | *bla*_SHV_-*bla*_TEM_-*bla*_CTX_ (1) | AMP (1), CRO (1), CZO (1), CXM (1), CTX (1), FEP (1) | IncHI1B (1) |
|  | K15K17K50K51K52-ST585 (1) | *iucA* (1) | 0 | 0 | 0 | 0 | *bla*_SHV_-*bla*_TEM_-*bla*_CTX_-*bla*_EBC_ (1) | GEN (1), AMP (1), CRO (1), CZO (1), CXM (1), CTX (1), FEP (1), CAZ (1), SAM (1) | IncHI1B (1) |
|  | K15K17K50K51K52-ST1336 (1) | *iucA*-*iroB* (1) | 1 | 0 | 1 | 0 | *bla*_SHV_ (1) | AMP (1), SXT (1) | IncF (1) |
|  | *wzi*568-ST2355 (1) | *iroB* (1) | 1 | 0 | 0 | 0 | *bla*_SHV_-*bla*_ACC_ (1) | AMP (1), CRO (1), CZO (1), CXM (1), CTX (1), FEP (1), LVX (1), SXT (1), SAM (1), AMC (1) | IncF-IncFII (1) |
|  | *wzi*582-ST1611 (1) | *iucA* (1) | 0 | 0 | 0 | 0 | *bla*_SHV_ (1) | AMP (1) | IncF (1) |
|  | *wzi*676-ST918 (1) | *iucA* (1) | 0 | 1 | 1 | 0 | *bla*_SHV_ (1) | AMP (1), SXT (1) | IncF-IncFII-IncHI1B (1) |
|  | NT^d^-ST17 (1) | *prmpA-iroB* (1) | 0 | 1 | 1 | 0 | *bla*_SHV_ (1) | AMP (1), SXT (1) | IncF (1) |
|  | NT^d^-ST152 (1) | *iucA* (1) | 1 | 0 | 0 | 0 | *bla*_SHV_ (1) | AMP (1), SXT (1) | IncF-IncFIB-IncFII-IncHI1B (1) |

^a^K type-ST, capsular genotype-sequence type.

^b^HM, hypermucoviscous phenotype; HSK, high resistance to serum; ESP, excessive siderophore production; HGML, high lethality in *G. mellonella* infection model.

^c^FOS, fosfomycin; GEN, gentamicin; AMK, amikacin; AMP, ampicillin; POL, polymyxin; CTX, cefotaxime; CZO, cefazolin; CRO, ceftriaxone; FEP, cefepime; FOX, cefoxitin; CAZ, ceftazidime; CXM, cefuroxime; LVX, levofloxacin; SXT, trimethoprim-sulfamethoxazole; TGC, tigecycline; SAM, ampicillin-sulbactam; TZP, piperacillin-tazobactam; AMC, amoxicillin-clavulanic acid; SCF, cefperazone-sulbactam; IPM, imipenem; MEM, meropenem; ETP, ertapenem.

^d^NT, non-typable.

Table S3. Information of randomly selected strains (n = 11) used in murine sepsis model.

|  | Strain^a^ | K type^b^ | ST^c^ | Setting^d^ (Wards) | Hypervirulence genes | | | | | | Resistance | | Hypervirulence phenotypes^e^ | | | | |
| --- | --- | --- | --- | --- | --- | --- | --- | --- | --- | --- | --- | --- | --- | --- | --- | --- | --- |
|  |  |  |  |  | *prmpA* | *prmpA2* | *iucA* | *iroB* | *peg344* | Carbapenemase | | HM | | SK (grade) | Su (%) | 24h-GML (%) |  |
| HvKp (n = 6) | 2020K105 | K1 | ST23 | CA (Gastroenterology) | + | + | + | + | + | - | | NEG | | 4 | 33.23 | 50 |  |
|  | 2019K134 | K2 | ST25 | HC (Neonatology) | + | - | - | + | + | NDM-1 | | POS | | 6 | 6.85 | 50 |  |
|  | 2020K14 | K2 | ST25 | HC (Neonatology) | + | - | - | + | + | - | | POS | | 5 | 0.13 | 90 |  |
|  | 2019K71 | K2 | ST86 | HC (Neonatology) | + | + | + | + | + | - | | POS | | 5 | 33.54 | 80 |  |
|  | 2019K136 | K2 | ST86 | CA (Emergency) | + | + | + | + | + | - | | POS | | 5 | 32.26 | 40 |  |
|  | 2020K202 | K16 | ST660 | CA (VIP) | + | + | + | + | + | - | | POS | | 2 | 25.97 | 70 |  |
| HgKp-Lv  (n = 5) | 2020K36 | K2 | ST25 | HC (Emergency) | + | - | + | + | + | - | | NEG | | 4 | 16.83 | 70 |  |
|  | 2020K102 | K16 | ST660 | HA (Neonatology) | + | + | + | + | + | - | | POS | | 2 | 13.38 | 30 |  |
|  | 2020K201 | KL47 | ST690 | CA (Pneumology) | - | + | + | + | - | KPC-2&  NDM-1 | | NEG | | 4 | 28.13 | 20 |  |
|  | 2020K74 | KL47 | ST11 | HA (Neonatology) | - | + | + | - | - | KPC-2 | | POS | | 4 | 22.26 | 0 |  |
|  | 2020K160 | wzi568 | ST2355 | HA (Neonatology) | - | - | + | - | - | - | | POS | | 4 | 1.00 | 0 |  |
| positive control | NTUH-K2044 | K1 | ST23 | CA | + | + | + | + | + | - | | POS | | 6 | 36.13 | 50 |  |
| negative control | cKP | NI^f^ | NI^f^ | CA (Pneumology) | - | - | - | - | - | - | | NEG | | 2 | - | 0 |  |

^a^Isolates were aligned according to the year of detection and serial number. Strains were selected with high-risk clones and sequence were randomly allocated by the RANDBETWEEN function in the Excel software, so potential confounders were inevitable system error. During group allocation, conduct of the experiment, outcome assessment, and data analysis, we followed the principle of double-blind.

^b^K type, capsular genotype.

^c^ST, sequence type.

^d^Setting for acusition: CA, community- acquired infection; HC, healthcare- associated infection; HA, hospital- acquired infection.

^e^HM, hypermucoviscous phenotype; SK, serum resistance; Su (%), siderophore units were defined as [(Ar – As)/Ar] × 100 = X%; 24h-GML, 24-hour lethality in *G. mellonella* infection model.

^f^NI, nonidentified.

Table S4. Characteristics comparison between hypervirulent *K. pneumoniae* (hvKp) (n = 27), hypervirulence genes positive *K. pneumoniae*-low virulence (hgKp-Lv) (n = 56) and classical *K. pneumoniae* (cKp) (n = 269) strains

|  | **hvKp (n = 27)** | **hgKp-Lv (n = 56)** | **cKp (n = 269)** | ***P* Value^a^** |
| --- | --- | --- | --- | --- |
| **Demographic data** |  |  |  |  |
| Age (yrs) (median [IQR]^b^) | 3.95 (0.25 - 9.86) | 0.12 (0.05 - 0.82) | 0.67 (0.08 - 3.00) | <.0001* (Z = 21.5962) |
| Male sex (n [%]^b^) | 18 (66.7) | 32 (57.1) | 156 (58.0) | 0.6660 (χ^2^ = 0.8128) |
|  |  |  |  |  |
| **Laboratory examination** |  |  |  |  |
| White blood cell count (×10^9^/L) (median [IQR]^b^) | 8.03 (5.76 - 13.65) | 11.41 (7.86 - 18.72) | 10.51 (7.49 - 14.20) | 0.0802 (Z = 5.0454) |
| Neutrophil ratio (%) (median [IQR]^b^) | 51.40 (33.50 - 64.70) | 48.70 (31.75 - 65.20) | 45.65 (30.50 - 68.80) | 0.9648 (Z = 0.0716) |
| C-reactive protein (mg/L) (median [IQR]^b^) | <=5 (<=5 - 10) | 5.5 (<=5 - 14.5) | <=5 (<=5 - 16) | 0.8136 (Z = 0.4126) |
| Procalcitonin (ng/ml) (median [IQR]^b^) | 0.16 (0.08 - 0.44) | 0.29 (0.14 - 0.94) | 0.21 (0.08 - 0.74) | 0.3671 (Z = 2.0042) |
| Direct bilirubin (DB) (umol/L) (median [IQR]^b^) | 2.20 (1.40 - 4.30) | 6.64 (1.70 - 14.10) | 4.10 (1.82 - 9.44) | 0.0099* (Z = 9.2250) |
| Total bilirubin (TB) (umol/L) (median [IQR]^b^) | 8.07 (4.89 - 12.16) | 35.01(8.01 - 88.42) | 11.20 (5.94 - 44.02) | 0.0017* (Z = 12.7132) |
| Alanine aminotransferase (U/L) (median [IQR]^b^) | 18.50 (13.00 - 28.00) | 20.00 (13.00 - 38.00) | 18.00 (11.00 - 39.00) | 0.8993 (Z = 0.2123) |
| Aspartate aminotransferase (U/L) (median [IQR]^b^) | 30.50 (23.00 - 49.00) | 35.50 (24.00 - 66.00) | 39.50 (26.00 - 55.00) | 0.3311 (Z = 2.2104) |
| Creatinine (umol/L) (median [IQR]^b^) | 25.50 (18.00 - 41.00) | 24.00 (18.00 - 35.00) | 23.00 (18.00 - 32.50) | 0.8341 (Z = 0.3627) |
| Uric acid (UA) (umol/L) (median [IQR]^b^) | 262.50 (168.00 - 320.00) | 160.00 (117.00 - 216.00) | 180.50 (128.00 - 250.00) | 0.0335* (Z = 6.7931) |
| Total protein (TP) (g/L) (median [IQR]^b^) | 66.57 (56.61 - 71.27) | 53.84 (47.08 - 60.36) | 57.11 (50.45 - 65.56) | 0.0014* (Z = 13.1027) |
| Albumin (ALB) (g/L) (median [IQR]^b^) | 42.30 (39.01 - 46.78) | 35.39 (32.85 - 38.95) | 38.30 (34.30 - 42.12) | 0.0002* (Z = 16.9502) |
| Age × DB (yrs × umol/L) (RR [95% CI])^c^ | 1.946E-013 (1.000E-013 - 90252974961) | 2.162 (0.086 - 54.208) | - | 0.287^d^; 0.639^e^ |
| Age × TB (yrs × umol/L) (RR [95% CI])^c^ | 0.779 (0.085 - 7.179) | 1.780 (0.001 - 2821.337) | - | 0.878^d^; 0.826^e^ |
| Age × UA (yrs × umol/L) (RR [95% CI])^c^ | 0.474 (0.161 - 1.396) | 0.767 (0.273 - 2.157) | - | 0.615^d^; 0.176^e^ |
| Age × TP (yrs × g /L) (RR [95% CI])^c^ | 75.489 (0.000 - 11413160.27) | 0.260 (2.875E-006 - 23563.884) | - | 0.817^d^; 0.477^e^ |
| Age × ALB (yrs × g /L) (RR [95% CI])^c^ | 0.000 (2.147E-013 - 93095.287) | 0.033 (7.340E-011 - 14515700.52) | - | 0.736^d^; 0.383^e^ |

^a^Test statistics are in brackets, *P* < 0.05(*).

^b^Values are presented as median (25th - 75th percentile) or No. (%) of cases.

^c^Values are presented as risk ratio (95% confidence interval) using multinomial logistic regression enrolled in age, DB, TB, UA, TP, ALB, age × DB, age × TB, age × UA, age × TP and age × ALB.

^d^*P* values were based on the comparison between hvKp group and cKp group.

^e^*P* values were based on the comparison between hgKp-Lv group and cKp group.
