# Supplementary material for: Nosocomial dissemination of hypervirulent Klebsiella pneumoniae with high-risk clones among children in Shanghai
Source: Front Cell Infect Microbiol. 2022 Aug 29;12:984180. doi: 10.3389/fcimb.2022.984180 (PMC9464974; doi:10.3389/fcimb.2022.984180)
Supplement: Supplementary file 2 [file DataSheet_1.pdf]

Supplementary-Figures

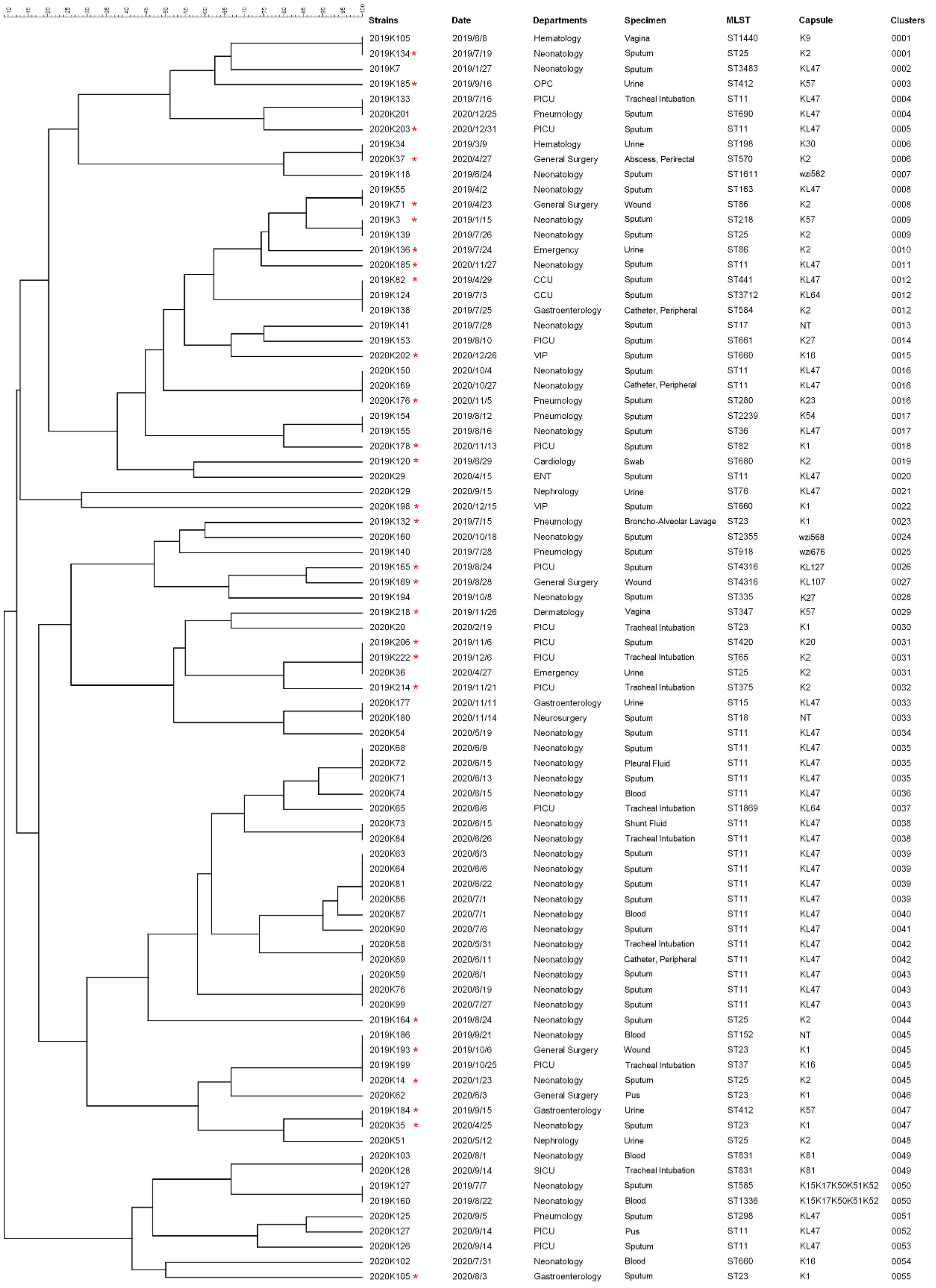

FIGURE S1 Phylogenetic tree of hypervirulence genes positive *K. pneumoniae* (hgKp) isolates (n = 83). The evolutionary analysis for finger printing profiles of Intergenic Consensus-Polymerase Chain Reaction (ERIC-PCR) was performed by BioNumerics software. Strains marked red "*" were hypervirulent *K. pneumoniae.*

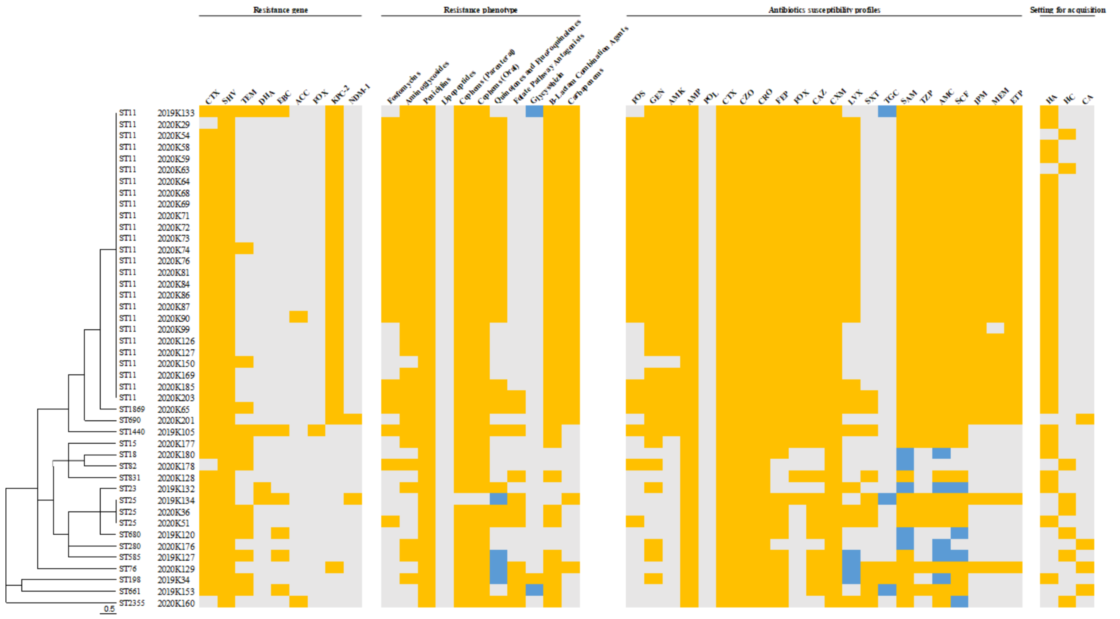

FIGURE S2 Characteristics of MDR-hgKp isolates (n = 44). Resistance gene (left), predicted phenotype (middle), and antibiotics susceptibility profiles (right) of ESBLs-producing isolates (n = 44) (from top to bottom are isolates in the phylogenetic relationship analyzed by Phyloviz 2.0 software using the hierarchical clustering algorithm based on the complete-linkage method of hamming distance between STs). Yellow filling means resistant, blue means intermediate, and grey means susceptible. FOS, fosfomycin; GEN, gentamicin; AMK, amikacin; AMP, ampicillin; POL, polymyxin; CTX, cefotaxime; CZO, cefazolin; CRO, ceftriaxone; FEP, cefepime; FOX, cefoxitin; CAZ, ceftazidime; CXM, cefuroxime; LVX, levofloxacin; SXT, trimethoprim-sulfamethoxazole; TGC, tigecycline; SAM, ampicillin-sulbactam; TZP, piperacillin-tazobactam; AMC, amoxicillin-clavulanic acid; SCF, cefperazone-sulbactam; IPM, imipenem; MEM, meropenem; ETP, ertapenem; HA, hospital-associated infection; HC, healthcare- associated infection; CA, community- associated infection.

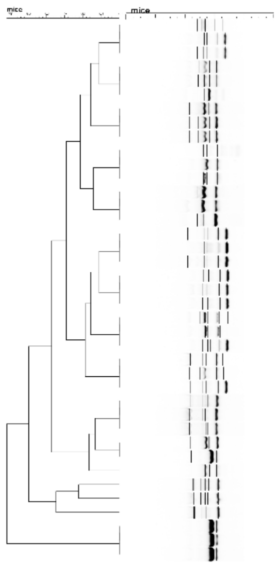

Key	72h survival rate	Genotype	Virulence genes
2020K102-1	5/5	K16-ST660	prmpA, prmpA2, iucA, iroB, peg344
2020K102-2	5/5	K16-ST660	prmpA, prmpA2, iucA, iroB, peg344
2020K102-3	5/5	K16-ST660	prmpA, prmpA2, iucA, iroB, peg344
2020K105-1	0	K1-ST23	prmpA, prmpA2, iucA, iroB, peg344
2020K105-2	0	K1-ST23	prmpA, prmpA2, iucA, iroB, peg344
2020K105-3	0	K1-ST23	prmpA, prmpA2, iucA, iroB, peg344
NTUH-K2044-1 (positive control)	0	K1-ST23	prmpA, prmpA2, iucA, iroB, peg344
NTUH-K2044-2 (positive control)	0	K1-ST23	prmpA, prmpA2, iucA, iroB, peg344
NTUH-K2044-3 (positive control)	0	K1-ST23	prmpA, prmpA2, iucA, iroB, peg344
2020K160-1	5/5	wzi568-ST2355	iroB
2020K160-2	5/5	wzi568-ST2355	iroB
2020K160-3	5/5	wzi568-ST2355	iroB
2020K38-1 (negative control)	5/5	NT	none
2020K38-2 (negative control)	5/5	NT	none
2020K38-3 (negative control)	5/5	NT	none
2019K136-1	0	K2-ST86	prmpA, prmpA2, iucA, iroB, peg344
2019K136-2	0	K2-ST86	prmpA, prmpA2, iucA, iroB, peg344
2019K136-3	0	K2-ST86	prmpA, prmpA2, iucA, iroB, peg344
2019K71-1	0	K2-ST86	prmpA2, iucA
2019K71-2	0	K2-ST86	prmpA2, iucA
2019K71-3	0	K2-ST86	prmpA2, iucA
2020K201-1	5/5	KL47-ST690	prmpA2, iucA, iroB
2020K201-3	5/5	KL47-ST690	prmpA2, iucA, iroB
2020K201-2	5/5	KL47-ST690	prmpA2, iucA, iroB
2020K36-1	5/5	K2-ST25	prmpA, iucA, iroB, peg344
2020K36-2	5/5	K2-ST25	prmpA, iucA, iroB, peg344
2020K36-3	5/5	K2-ST25	prmpA, iucA, iroB, peg344
2019K134-1	0	K2-ST25	prmpA, iroB, peg344
2019K134-3	0	K2-ST25	prmpA, iroB, peg344
2019K134-2	0	K2-ST25	prmpA, iroB, peg344
2020K74-1	4/5	KL47-ST11	prmpA2, iucA
2020K74-2	4/5	KL47-ST11	prmpA2, iucA
2020K74-3	4/5	KL47-ST11	prmpA2, iucA
2020K14-2	0	K2-ST25	prmpA, iroB, peg344
2020K14-3	0	K2-ST25	prmpA, iroB, peg344
2020K14-1	0	K2-ST25	prmpA, iroB, peg344
2020K202-1	3/5	K16-ST660	prmpA, prmpA2, iucA, iroB, peg344
2020K202-2	3/5	K16-ST660	prmpA, prmpA2, iucA, iroB, peg344
2020K202-3	3/5	K16-ST660	prmpA, prmpA2, iucA, iroB, peg344

FIGURE S3 Homology of randomly selected strains (n = 11) used to build murine sepsis model. Strains-1, strains before mice injection; Strains-2 and Strains-3, strains recovered from a separate portion of the harvested livers of the injected mice. Fresh livers were grinded in paraformaldehyde-free PBS. Bacteria in supernatant were cultured on blood agar plate at 37 °C overnight, and identified by MALDI-TOF mass spectrometry. The evolutionary analysis for finger printing profiles of Intergenic Consensus-Polymerase Chain Reaction (ERIC-PCR) was performed by BioNumerics software.

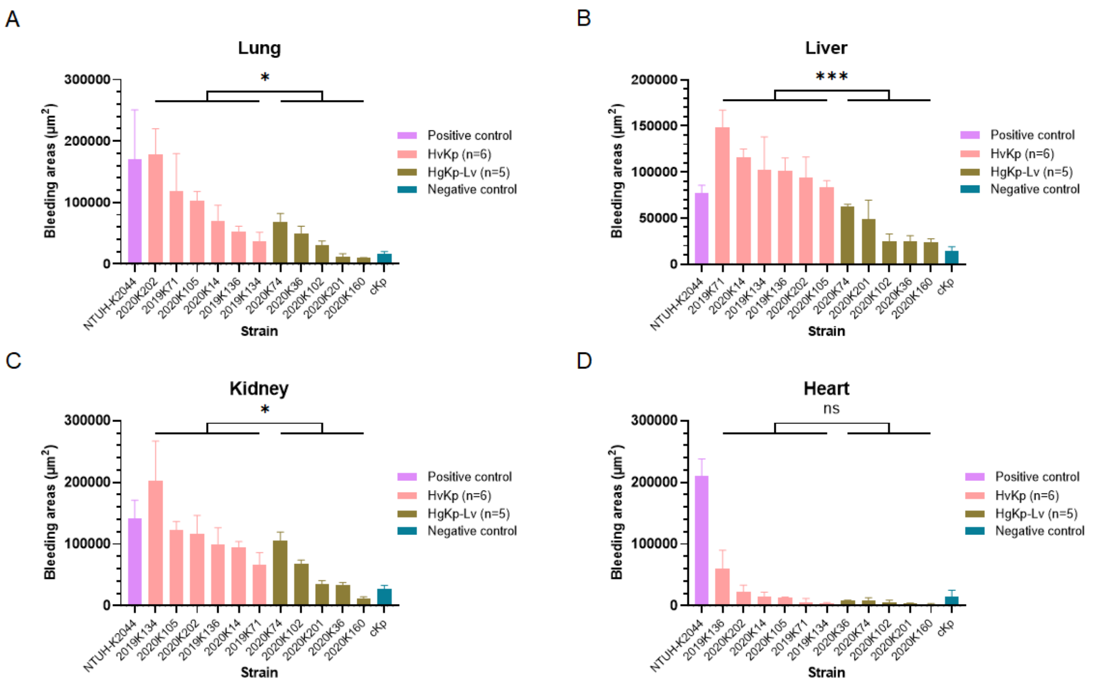

FIGURE S4 Damage of hvKp infection to mice. Data of organs bleeding areas were expressed as means ± SDs, $P < 0.05$ (*), $P < 0.001$ (***) and $P > 0.05$ (ns). Between hvKp and hgKp-Lv isolates infected mice, significant differences were observed in the bleeding areas of lung (t = 2.33, $P = 0.0446$, 95% confidence interval [CI] = 42018 to 98612), of liver (t = 5.62, $P = 0.0003$, 95% CI = 1776.2 to 116974), of kidney (t = 2.58, $P = 0.0298$, 95% CI = 8126.2 to 124402), but not of heart (t = 1.66, $P = 0.1558$, 95% CI = -7219 to 35917) using independent-samples t-test.

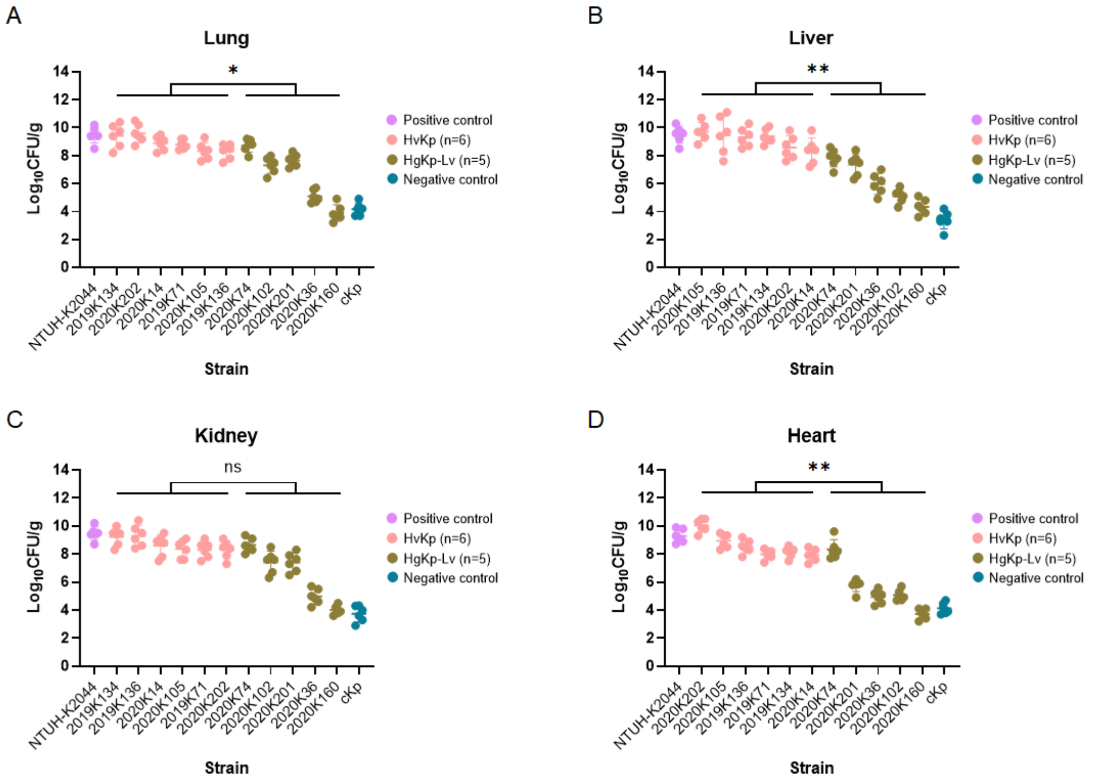

FIGURE S5 Bacterial burdens in the mice organs. Data shown are means ± SDs, $P < 0.05$ (*), $P < 0.01$ (**) and $P > 0.05$ (ns). Between hvKp and hgKp-Lv isolates infected mice, significant differences were observed in bacterial CFU of lung ($t = 2.81$, $P = 0.0205$, 95% confidence interval [CI] = 6.6 to 9), of liver ($t = 4.64$, $P = 0.0012$, 95% CI = 6.5 to 9), of heart ($t = 3.87$, $P = 0.0038$, 95% CI = 5.9 to 8.5), and a slight difference was observed of kidney ($t = 2.53$, $P = 0.0596$, 95% CI = 6.5 to 8.8) using independent-samples t-test.
